# Bisphenol A induces coronary endothelial cell necroptosis by activating RIP3/CamKII dependent pathway

**DOI:** 10.1038/s41598-020-61014-1

**Published:** 2020-03-06

**Authors:** P. Reventun, S. Sanchez-Esteban, A. Cook, I. Cuadrado, C. Roza, R. Moreno-Gomez-Toledano, C. Muñoz, C. Zaragoza, R. J. Bosch, M. Saura

**Affiliations:** 1 Biology systems Dpt, University Alcalá (UAH), Madrid, Spain; 2 Pharmacology, Pharmacognosy and Botanics Dpt, Complutense University (UCM), Madrid, Spain; 3 Joint Unit of Cardiovascular Research University Francisco de Vitoria and Hospital Ramon y Cajal, Madrid, Spain

**Keywords:** Cardiac hypertrophy, Cardiac hypertrophy

## Abstract

Epidemiological studies link long term exposure to xenoestrogen Bisphenol-A to adverse cardiovascular effects. Our previous results show that BPA induces hypertension by a mechanism involving CamKII activation and increased redox stress caused by eNOS uncoupling. Recently, CamKII sustained activation has been recognized as a central mediator of programmed cell death in cardiovascular diseases, including necroptosis. However, the role of necroptosis in cardiac response to BPA had not yet been explored. Mice exposed to BPA for 16 weeks showed altered heart function, electrical conduction, and increased blood pressure. Besides, a stress test showed ST-segment depression, indicative of cardiac ischemia. The hearts exhibited cardiac hypertrophy and reduced vascularization, interstitial edema, and large hemorrhagic foci accompanied by fibrinogen deposits. BPA initiated a cardiac inflammatory response, up-regulation of M1 macrophage polarization, and increased oxidative stress, coinciding with the increased expression of CamKII and the necroptotic effector RIP3. In addition, cell death was especially evident in coronary endothelial cells within hemorrhagic areas, and Evans blue extravasation indicated a vascular leak in response to Bisphenol-A. Consistent with the *in vivo* findings, BPA increased the necroptosis/apoptosis ratio, the expression of RIP3, and CamKII activation in endothelial cells. Necrostatin-1, an inhibitor of necroptosis, alleviated BPA induced cardiac dysfunction and prevented the inflammatory and hemorrhagic response in mice. Mechanistically, silencing of RIP3 reversed BPA-induced necroptosis and CamKII activation in endothelial cells, while inhibition of CamKII activation by KN-93 had no effect on RIP3 expression but decreased necroptotic cell death suggesting that BPA induced necroptosis is mediated by a RIP 3/CamKII dependent pathway. Our results reveal a novel pathogenic role of BPA on the coronary circulation. BPA induces endothelial cell necroptosis, promotes the weakening of coronary vascular wall, which caused internal ventricular hemorrhages, delaying the reparative process and ultimately leading to cardiac dysfunction.

## Introduction

Bisphenol A (BPA) is a ubiquitously used chemical with endocrine-disrupting activity. Many of BPA effects are dependent on its ability to interact with estrogen receptors^1,2^. Due to its widespread use, humans are exposed continuously to accumulative doses of BPA. Significant exposure often occurs by consuming food and beverages contaminated with BPA and in dental procedures, so that up to 92.6% of the human population has detectable BPA levels in their bodies^3,4^. Epidemiological studies have demonstrated that higher BPA urine concentrations are associated with increased morbidity and mortality from cardiovascular diseases^5,6^, and several studies in humans and animal models have suggested a causative role for BPA in cardiovascular disease including hypertension, atherosclerosis, cardiac hypertrophy and arrythmias^7–10^. Recently, it has been reported that a life long exposure to BPA in rodents produces arrhythmogenic effects^11^. Moreover, it has been demonstrated that acute or chronic exposure to BPA, impaired functional recovery after myocardial infarction due to a reduced ability to induce macrophage polarization from proinflammatory to a reparative state^12,13^. Indeed, there is an increasing concern about BPA higher bioaccumulation in developing organisms and special vulnerable populations such as patients exposed to BPA leaching from medical devices, people handling thermal paper receipts, or plastic industry workers^4,13,14^.

Despite all the evidence, there is not a consensus about the extent of cardiac damage caused by BPA. Moreover, the mechanisms by which BPA exposure induces cardiovascular disease remains to be fully elucidated. Many reports rely on cardiomyocyte-specific mechanisms to explain the adverse cardiac effects of BPA. So, most studies reported alterations on electrical conduction^15,16^, dysregulation of the contractile apparatus^17,18^, and metabolic abnormalities on cardiomyocytes^19^, but little attention has been focused on the coronary circulation; indeed, BPA can promote the development of atherosclerosis^20^. A connection between urinary BPA and hypertension has been established in human and animal models, and the role of BPA in peripheral disease has been identified^8,21^. Recently, it has been reported that although BPA itself does not affect the coronary response in coronary ischemia/reperfusion, it reduces coronary flow after reperfusion, and when administered together with estrogens, it counteracts their protective effect during reperfusion^22^.

Our previous studies demonstrated a direct effect of orally administered BPA in mice, causing hypertension and endothelial dysfunction due to endothelial nitric oxide synthase (eNOS) uncoupling via Angiotensin II/Ca^2+^-CamKII pathway^23^. In the heart, calmodulin-dependent protein kinase II (CamKII) regulates cardiomyocyte Ca^2+^ handling, contractility, and cell survival; however, sustained CamKII activation is recognized to promote heart failure^24^, arrhythmia^25^, and sudden cardiac death^26^. Besides, chronic CamKII activation contributes to endothelial-dependent vascular disease in diabetes and hypertension, which can also impact cardiac function. Recently, CamKII sustained activation has been recognized as a central mediator of programmed cell death, including necroptosis, in cardiovascular diseases^27^. CamKII can be a substrate of RIP 3, the main switch in necroptosis, during ischemia-reperfusion resulting in mPTP opening and cardiac cell death^28^. However, the role of necroptosis in cardiac response to BPA had not yet been explored.

In this study, we sought to evaluate if chronic administration of BPA had any effect on cardiac function. Since BPA induced cardiac damage might be secondary to vascular effects, here we explore whether BPA effects are direct, on cardiomyocytes, or indirect, on coronary vascular endothelium. In addition, the underlying molecular mechanism of these effects will be explored. Clarifying the relationships between BPA and coronary vascular function and the molecular mechanism involved in cardiac cell death may widen our understanding of BPA’s impact on cardiovascular disease.

## Material and Methods

A detailed listing of the reagents and antibodies used through the study are provided in Supplementary Data.

### Animals

Wild-type CD1 mice were purchased from Charles River (Wilmington, MA, USA) and housed in our animal facilities with four mice/cage located in isolated rooms. All animal procedures were approved by the University of Alcala Animal Care Committee and Autonomous Community of Madrid (experimental procedure 007/16) and conformed to the EU Directive regarding the protection of animals used for experimental and other scientific purposes (enacted under Spanish law 1201/2005). CD1 male mice of 8 weeks (~30 g weight) were used.

Ethanol dissolved-BPA was added to the drinking water at final concentration of 4 × 10^−5^ M. CT consisted of an equivalent volume of ethanol (final concentration 0.01%) in drinking water. This value delivers BPA ≤ 50 mg/kg animal weight/day considered a *low dose*^29^. Only male mice were included in our study.

To study the BPA effects on cardiac necroptosis, we randomly divided mice into 2 groups: necrostatin- 1 (NEC) or vehicle control group (CT). Some were administered BPA as above for 4 to 8 weeks (BPA + NEC and NEC). Necrostatin-1 was injected intraperitoneally 3 mg/kg animal weight/day, 3 days a week, as reported elsewhere^30^.

### Cell cultures

Murine aortic ECs (MAECs) were isolated from mouse aorta, as previously reported^31^. Briefly, the aortas were sectioned into 2-mm pieces, deposited in Matrigel solution, and fed with fresh growth medium for seven days [DMEM/HAM´s medium, 20% FBS, 0.05 mg/mL penicillin/streptomycin, and 2.5 μg/mL amphotericin]. The tissue was removed, and 500 μL of Cell recovery solution was added to each culture. The Matrigel layer was removed and poured on ice for one hour. The solution was centrifuged at 4 °C, resuspended in 4 mL of growing medium, and plated. MAECs were selected by their ability to express the intercellular adhesion molecule-2 (ICAM-2) protein and purified with a flow cytometry cell sorter (DAKO)^32^. Purification was verified by confocal microscopy of MAECs double-stained with Von Willebrand factor antibodies.

H9c2 cells, a myoblastic cell line derived from embryonic rat myocardium, were cultured in the L-glutamine Dulbecco’s modified Eagle’s medium containing 10% fetal bovine serum and 0.05 mg/mL penicillin/streptomycin in a humidified CO_2_ incubator with 5% CO2 at 37 °C^33^.

### Cardiac myocytes and non-myocytes Isolation from the mouse heart

In order to elucidate the effect of BPA in mouse coronary endothelial cells, cardiac myocytes and non-myocytes cellular fractions were isolated from the mouse heart following the method described by Ackers-Johnson M, *et al*.^34^. Briefly, hearts from CT and BPA mice were perfused with a high EDTA buffer, excised from the circulatory system, and transferred to a 60 mm plate containing EDTA buffer. Digestion was achieved by injection of collagenase solution into the left ventricle (LV). When digestion was observed, the atria and right ventricle were carefully removed, and the left ventricle mechanically dissociated. Enzimatic activity was blocked with stop buffer and the cell suspension was passed through a 100-μm filter, followed by four sequential rounds of gravity settling.

The cell pellet in each round was enriched with myocytes and ultimately formed a highly pure myocyte fraction, whereas the supernatant from each round was combined to produce a fraction containing non-myocyte cardiac populations. Non-myocytes cells were characterized by immunofluorescence staining of the endothelial marker CD31 and α-smooth muscle actin. CD31 positive cells quantification was performed, counting six different homogeneous fields that contain a mean of 60 cells/field. Endothelial cells represents the 73 ± 1.23% of the nonmyocytes fraction. Myocytes and non-myocytes fraction were lysed using protein lysis buffer and analyzed by western blotting.

### ECG recordings

Mice were anesthetized with isoflurane (2% in pure O2). Fine needle electrodes (25 G) were inserted subcutaneously at the level of both armpits and left groin and connected to an AC amplifier (Cyberamp, Axon Instruments). The ECG leads were placed initially in the lead II configuration and exchanged when required, to the lead I and lead II at the pre-amplifier. The signals were amplified 500 times and band-pass filtered between 1 Hz and 100 Hz, digitized at 1000 Hz (Power 1401, CED, UK) and stored for off-line analysis using Spike 2 software (CED, UK)^35^.

### Echocardiography

Mouse hearts were visualized by echocardiography by using a Vivid Q ultrasound system (GE healthcare) equipped with a 12.5 MHz scan head. Mice were anesthetized with 1.5% isoflurane gas, resulting in a heart rate of approximately 400 beats/min. Parasternal short-axis-view images of the heart were recorded in a B-mode to allow M-mode recordings by positioning the cursor in the parasternal short-axis view perpendicular to the interventricular septum and posterior wall of the left ventricle^36^. From these recordings, the following parameters were determined using the on-site software cardiac package: systolic and diastolic Interventricular septum thickness (IVS), systolic and diastolic left-ventricle internal diameter (LVID), systolic and diastolic left-ventricle posterior Wall thickness (LVPW), left-ventricle ejection fraction (EF), left ventricle shortening fraction (FS), heart rate (HR), and cardiac output (CO).

### Dobutamine stress test

Mice were injected a maximally effective dose of the β -AR agonist, dobutamine (3 μg/g)^37^. Inotropic and chronotropic responses to this stimulation were verified in all mice by echocardiography and ECG recordings of DI and II for 15 min.

### Blood pressure

Indirect measurements of blood pressure were obtained in conscious animals using a tail-cuff sphygmomanometer (LE 5001 Pressure Meter; Letica Scientific Instruments, Hospitalet, Spain)^23^. The animals were trained for 5 d before starting the measurement to prevent stress and were pre-warmed to 30 °C with a heater (LE5660/6, Letica Scientific Instruments, Hospitalet, Spain). Arterial pressure was measured several times between 9:00 and 12:00 AM, and pressure values were considered acceptable at ten consecutive measurements.

### Vascular permeability assay

Evans blue is vital dye used to study blood vessel and cellular membrane permeability^38^. Evans blue was injected IP, 24 hours before euthanization, to allow circulation. The striking blue color can be identified the EBD-albumin conjugate within the tissue *ex-vivo*^39^. To extract Evans blue dye, 250 µL deionized formamide was added to the dry tissue and incubated overnight in an oven or a heating block at 55 °C, then loaded into a 96-well plate for absorbance reading with a spectrophotometer. Evans blue was also detected by confocal microscopy of the LV sections due to the fluorescence spectrum of this product (620 nm excitation/680 nm emission)^40^.

### Histology

LV sections were fixed in a 10% formalin solution, dehydrated in ethanol, and then embedded in paraffin as previously described^36^. Tissue sections (5 μm) were obtained in a microtome, were deparaffinized, rehydrated, and stained with Masson’s trichrome staining kit (EMD Millipore Corporation, Billerica, MA, USA) and Sirius red staining (Sigma- Aldrich, San Luis, MI, USA) for fibrosis quantification. Hematoxylin/Eosin staining was performed to explore heart morphology (Sigma-Aldrich, San Luis, MI, USA).

### Immunoblot

Protein lysates were immunoblotted as previously described^41^. Twenty-five μg of total protein was separated in a 10% SDS-polyacrylamide gel electrophoresis. For protein detection, blocked membranes were incubated with specific antibodies, washed, and incubated with a secondary antibody. The bands were visualized with the Super-Signal detection kit (Pierce, Waltham, MA, USA).

### Immunohistochemistry

Samples were boiled in retrieval buffer for 20 min after xylene deparaffinization. Master polymer plus detection system (Master Diagnostica, Madrid, Spain) was used, and antibody incubation was overnight at 4 °C. Sections were incubated with secondary antibodies for 1 hour at room temperature and counterstained with Harry´s hematoxylin, dehydrated and mounted with DPX (Casa Alvarez, Madrid, Spain)^42^. Images obtained of at least five different hearts per condition (6 per animal) were taken for data quantification using bright-field microscope (Eclipse 50i; Nikon, Tokyo, Japan).

### Confocal microscopy

Slides containing tissue sections were incubated with the primary antibodies overnight 4 °C. After washing with PBS, the slides were incubated with FITC, Alexa-488, or Alexa-647-conjugated secondary antibodies for 1 hour at room temperature. Nuclei were stained with Hoechst. Images were taken for data quantification using a Leica TCS SP5 confocal microscope (*UAH-NANBIOSIS-CIBER-BNN)*. At least five different fields per condition were obtained.

### Analysis of capillaries densities in the myocardium

For blood vessel counting heart sections obtained from CT and BPAtreated mice were stained with CD31 antibody as described previously^43^. Sections (6 μm) were blocked with 5% BSA for 30 min and, incubated with CD31antibody, a specific marker of endothelial cells overnight at 4 °C and the secondary antibody Alexa fluor 647 was added for 1 hour, room temperature light protected. The samples were incubated with FITC–conjugated wheat germ agglutinin (WGA) (Life Technologies, Carlsbad, Ca, USA) to delimit cardiomyocyte plasma membrane for 1 hour and Hoechst was added to localized the nuclei. Images of subepicardial regions of the left ventricular (LV) wall and the interventricular septum (IVS) on the section were obtained by confocal microscopy (Leica TCS SP5). Positive CD 31 endothelial cells were quantified from at least six random high-power fields from different heart regions (left ventricle, right ventricle, and septum) by a blinded investigator. The capillary count was analyzed with Fiji^44^. The results were presented as capillary density per field for each heart region analyzed.

### Cell transfection

MAECs were transfected with 50 nM non-targeting siRNA(si-Scramble) or RIP 3-targeting siRNA (si-RIP 3) (Santa Cruz Biotech, Santa Cruz, CA, USA)^45^ using Lipofectamine 2000 transfection reagent and Opti-MEM (Gibco, Waltham, Ma, USA) for 6 hour. Following 24 hours transfection, cells were incubated with BPA in 5% serum DMEM culture medium for 24 hours at 37 °C, 5% CO2.

### Survival, apoptosis and necrosis assay

An MTT assay was used to measure cell viability^46^. Briefly, MAEC (6000 cells/well) were seeded in 24-well plates overnight and were then incubated with various concentrations of BPA at 37 °C for 24 h. MTT (0,5 mg/mL) was added to each well. After three hours incubation, supernatants were removed, and 150 µL DMSO was added to each well as a solvent. Absorbance was measured at 492 nm in a spectrometer plate reader (ELx800, Bio-Tek Instruments, Winooski, VE, USA). Non treated cells (control group) were regarded as having 100% viability.

To determinate cell death in tissue, we use DeadEnd™ Fluorometric TUNEL System (Promega, Madison, WI, USA) following the manufacturer instructions, in combination to Polyclonal Rabbit Anti-FITC/HRP, the stain was developed by addition of DAB substrate (Dako, Santa Clara, CA, USA). Samples were counterstained with Mayer’s hematoxylin and mounted with DPX and visualized in a bright field microscope (Eclipse 50i; Nikon, Tokyo, Japan). Images (2 sections, n = 5 animals/group). Five slide fields were randomly examined using a defined rectangular area under 40 × magnifications. Apoptotic cells were counted under 400 × magnifications. Results are expressed as the percentage of TUNEL-positive cells versus the total number of cell nuclei per field^47^.

Necrosis and apoptosis determination by flow cytometry were performed in cells treated with BPA and silenced for RIP3 or a siRNA scramble as control. The cells were trypsinized and resuspended in 150 μl of 1 × binding buffer. Then 5 μl of 20 μg/mL Annexin V-FITC conjugated and 5 μl of propidium iodide (PI) were added and cells incubated for 15 minutes in the dark at room temperature according to manufacturer´s instructions. Cells were analyzed by FACS Calibur (Becton, Dickinson Company, NJ, USA). The percentages of cells in each quadrant were analyzed using Cyflogic software (Cyflo Ltd, Turku, Finland). Results of flow cytometry were obtained by calculating the number of necrotic cells (PI^+^/Annexin^−^) and apoptotic cells (PI^−^/Annexin^+^). Double negative were considered live cells and annexin V^+^-PI^+^ were late apoptotic or necroptotic cells. All the results experiments were performed in duplicate and repeated at least three times.

### mRNA expression

Total RNA from cardiac tissue was extracted with TRIzol reagent from Invitrogen Corporation (Carlsbad, CA, USA) following the manufacturer´s instructions. First-strand cDNA was synthesized from 2 μg of total RNA in a 20 μl reaction mixture using the High Capacity cDNA reverse transcription kit, and the qPCR reaction was performed with SYBR select master mix both of Life technologies (Carlsbad, CA, USA). The qPCR conditions and primers that were used are included in the Supplementary Information Table 1.

### Statistical analysis

Every experimental condition was duplicated within each experiment, and each experiment was repeated at least three times. For animal studies, n values refer to the number of individual animals used. Comparisons were made by analysis of variance, followed by Dunnett’s modification of the t-test when comparisons were made with common control and the unpaired two-tailed Student’s t-test for other comparisons. Results are expressed as mean ± SD, and differences were considered statistically significant at p < 0.05.

## Results

### BPA impaired cardiac function and induced cardiac hyperhrophy

**Figure 1:**
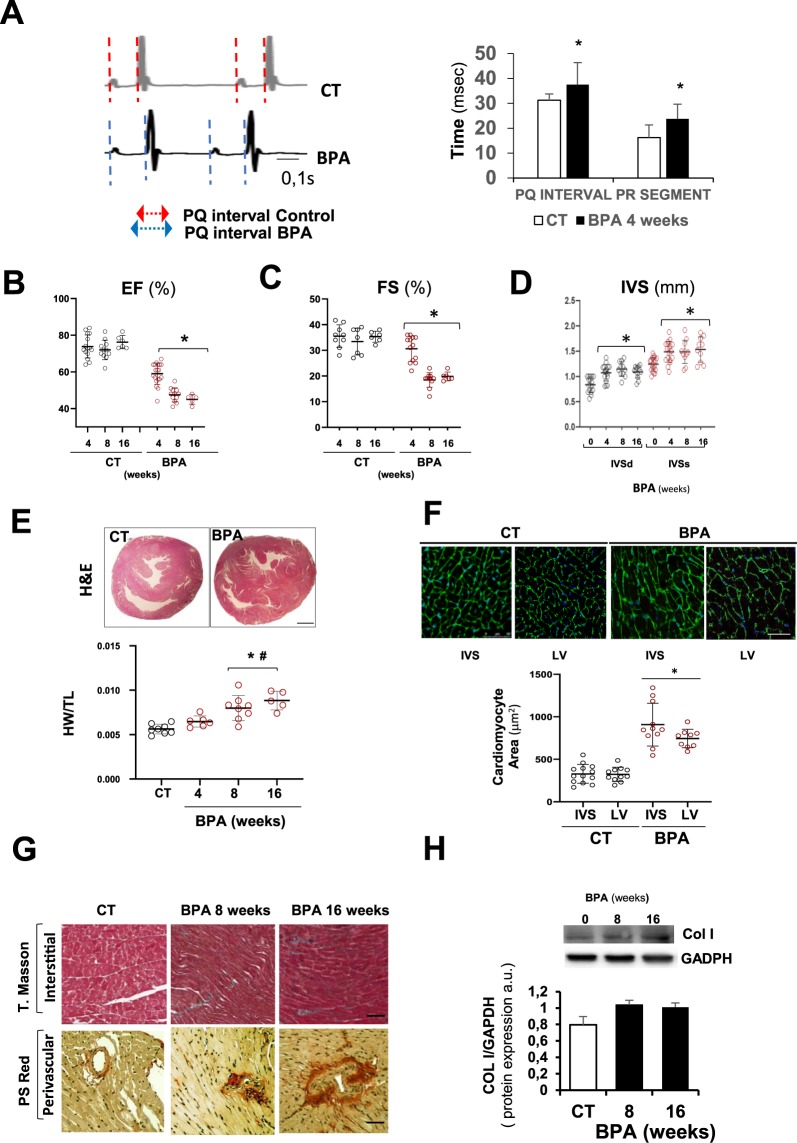
BPA induced impaired cardiac function and cardiac hypertrophy. ECG and echocardiography were performed in CT (vehicle treated) and BPA treated mice for 4, 8, and 16 weeks. (**A**) *Left panel:* Representative ECG recording in DII showing a longer PQ interval in 4 weeks BPA treated mice compared to CTmice. *Right panel* shows mean values for PQ interval and PR segment from ECGs recorded after 4 weeks of treatment (CT n = 10 and BPA n = 18, *p < 0.05). (**B**–**D**) shows LV ejection fraction (EF), Fractional shortening (FS) and interventricular septum thickness respectively (CT n = 12 and BPA n = 6–10) *p < 0.05 vs. CT; (**E**) *Upper panel:* Representative images of hematoxylin and eosin in heart sections from mouse after 16 weeks of BPA or CT showing IVS enlargement. Scale bar: 1000 μm. *Lower panel:* Quantification of heart weight to tibial length ratio (mg/mm) of CT and BPA treated mice at the indicated time points. (CT n = 12 and BPA n = 6–10 mice per group). *p < 0.05 vs. CT; # p < 0.05 vs. BPA 4 weeks (**F**) *Upper panel:* Representative images of wheat germ agglutinin (WGA)-fluorescein isothiocyanate-staining in mouse hearts after 16 weeks of treatment showing cardiac myocyte (CM) cross-sectional area at different heart regions (LV wall and interventricular septum, IVS). Scale bars: 20 μm. *Lower panel:* Quantitative data of CM hypertrophy cell surface area (n = 8–12 hearts per group with 300–600 CMs analyzed per heart). CM size was expressed as μm^2^. (**G**) Representative Masson Trichrome and Sirius red-stained sections of CT and BPA mice at 8 and 16 weeks showing perivascular fibrosis but not interstitial fibrosis in BPA treated mice. Scale bar = 60 µm. (**H**) Collagen type I protein expression measured by western blotting in whole heart tissue from CT and BPA treated mice. GADPH is used as loading control. The bar graph shows the average of n = 10 hearts per condition.

Previously, we had reported that oral administration of BPA at different concentrations induced hypertension in mice as early as five weeks starting at 4 × 10^−7^ M BPA and reaching a maximum effect at 4 × 10^−5^ M BPA^23^. We tested whether more prolonged exposure to BPA may negatively affect cardiac function. Mice were orally exposed to 4 × 10^−5^ M of BPA in the drinking water for 4, 8, and 16 weeks and cardiovascular functional abnormalities were examined. To assess the effect of BPA on cardiac conduction, we performed surface ECGs on CT (n = 10), and BPA treated mice (n = 18) (Fig. 1A). Interestingly, BPA treated mice displayed increased heart rate (526.25 ± 49.08 vs. CT = 440 ± 28.28; p < 0.001), and a significantly prolonged PQ interval and PR segment suggesting a first degree AV block.

Echocardiography analysis revealed that cardiac contractility was significantly impaired in BPA treated mice, as demonstrated by decreased ejection fraction (EF) **(**Fig. 1B**)** and fractional shortening (FS) (Fig. 1C). Besides, diastolic and systolic Interventricular septum thickness (IVSd) were increased, suggestive of cardiac hypertrophy **(**Fig. 1D**)**. Left ventricular posterior wall thickness was slightly, but not significantly, elevated. However, end-diastolic but especially end-systolic internal diameter was augmented in animals treated for 8 and 16 weeks with BPA **(**Supplementary Fig. S1A,B**)**. These results indicates that besides a contractile dysfunction, BPA also induced a slight increase in ventricular size, consistent with ventricular hypertrophy. As expected, BPA also increased systolic and diastolic blood pressure (BP) after 4 weeks, and, was further elevated at 16 weeks (Supplementary Fig S1C).

Consistent with the functional findings, the hearts were significantly enlarged after 16 weeks of BPA treatment, as detected by heart weight-to-tibial length ratio and hematoxylin and eosin sections **(**Fig. 1E**)**. Cardiomyocyte cross-sectional area measured by Wheat Germ Agglutinin (WGA) staining was also increased, especially at the interventricular septum and left ventricle wall, indicating cardiac hypertrophy **(**Fig. 1F**)**. Cardiac fibrotic remodeling was not found in BPA hearts when compared with CT mice **(**Fig. 1G **upper pannel)** and Col I expression was modestly increased in cardiac tissue at 8 and 16 weeks of BPA administration **(**Fig. 1H**)**. However, perivascular fibrosis was significantly increased after eight weeks of BPA **(**Fig. 1G **lower panel)**. Together these results indicate that BPA increased heart rate, impaired cardiac contractility, and induced cardiac hypertrophy.

### BPA induces cardiac ischemia under stress and chronic cardiac inflammation

**Figure 2:**
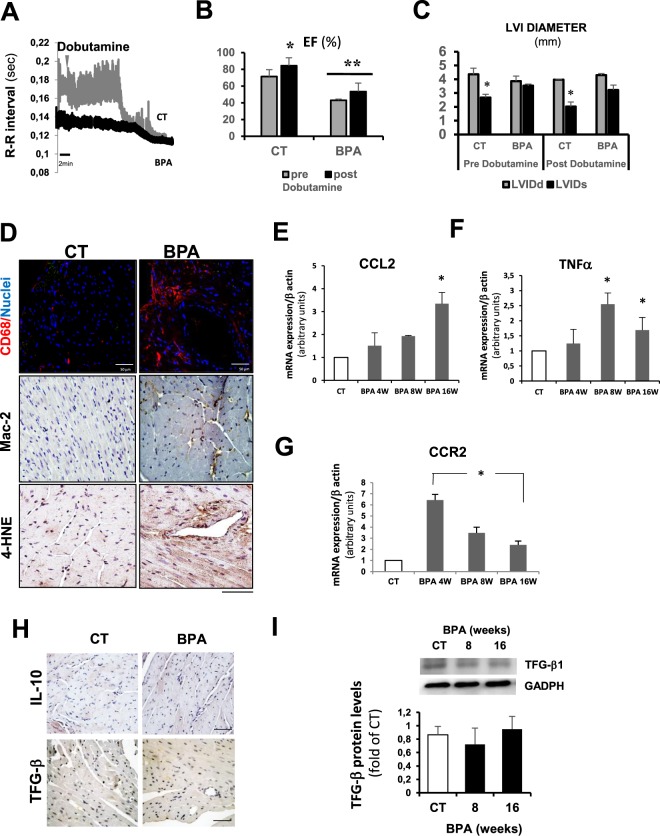
BPA induces cardiac ischemia and chronic inflammation. CT and 16 weeks BPA treated were challenged with an i.p. injection of 3μg/g of dobutamine while recording cardiac activity by EGC for 15 min. (**A**) Graphic representation of the RR interval of CT and BPA mice before and after dobutamine administration (n = 4–6 mice per group). (**B**,**C**) Echocardiogram analysis of CT and BPA mice before and after dobutamine administration showing Left ventricular ejection fraction (EF) and Left ventricular internal diameter (LVI Diameter) respectively. The data are presented as mean ± SD (n = 4 per group, EF: *p < 0.05 vs CT pre Dobu and **p < 0.05 vs BPA predobu; LVID *p < 0.05 vs LVIDd). (**D**) *Upper panel:* Representative confocal images showing infiltration of CD68^+^ macrophages (red) and nuclear staining with Hoetch (blue) as detected by confocal microscopy in heart sections from CT and 4 weeks treated BPA mice. *Middle and lower panels:* Immunohistochemistry in heart sections from CT and 8 weeks treated BPA mice detecting Mac-2 (macrophage marker), and 4-HNE (oxidative stress marker) at 16 weeks BPA (n = 8 for both groups, Scale bar = 60 µm). (**E**–**G**) RT-qPCR of CT and 4, 8, and 16 weeks BPA treated mice showing cardiac mRNA expression of: (**E**) CCL2 (MCP-1), (**F**) TNF-α and (**G**) CCR2 (CCL2 receptor) (n = 8 per condition with triplicates in each determination, *p < 0.05 vs. CT). (**H**) Images representative of immunostaining of IL10 and TFG-β1 in hearts from CT and 16 weeks treated BPA treated mice. Similar results were obtained in triplicate heart sections from n = 4 animals per condition. (**I**) TFG-β1 protein expression measured by western blotting in whole heart tissue from CT and BPA treated mice. The bar graph shows the average of n = 6 hearts per condition.

To test the pathophysiological implication of our findings, we performed a dobutamine stress echocardiography study in our BPA treated mice. Following administration of dobutamine (DB), heart rate (HR) increased significantly from baseline values in CT mice, but not in BPA treated mice, suggesting a BPA-mediated impairment of chronotropic responsiveness to β-adrenergic stimulation (Supplementary Fig. S2A**)**. This effects was confirmed by the analysis of surface electrocardiogram in which shorter R-R intervals in response to DB challenge were observed only in CT mice, while R-R intervals failed to decrease further in BPA-treated mice when compared to BPA resting values **(**Fig. 2A**)**. Moreover, the depressed ST segment, indicative of cardiac ischemia, was evident in most animals treated with BPA, suggesting that the coronary reserve may also be compromised (Supplementary Fig. 2B). Additionally, a DB challenge markedly reduced cardiac contractility after 16 weeks of BPA, as evidenced by the inability to increase the EF **(**Fig. 2B**)**, the cardiac output **(**Supplementary Fig. S2C**)**, and the left ventricular diameter, compared with CT mice **(**Fig. 2C**)**. Altogether, BPA reported abnormal stress test results, which might be indicative of cardiac ischemia.

Cardiac inflammation plays a critical role in adverse cardiac remodeling, as observed in ischemic heart disease, cardiac hypertrophy, and heart failure^48^. Hematoxylin and eosin sections of cardiac tissue from BPA-treated mice revealed an inflammatory response as early as four weeks of treatment, in which localized foci of CD68^+^ and Mac-2^+^ macrophage infiltrates were detected. Increased oxidative stress as detected by 4-Hydroxynonenal (4-HNE) was also present after 16 weeks, which might contribute to BPA-induced myocardial injury (Fig. 2D).

Since mice after 16 weeks of BPA still presented significant inflammatory infiltrates, we explored whether BPA may prevent macrophage polarization toward a phenotype of inflammatory resolution. A qRT-PCR analysis showed that M1 markers (Tumor necrosis factor-alpha (TNF-α) and CCL2 **(**Fig. 2E,F**)** and Chemokine (C-C motif) ligand 7 (CCL-7), Chemokine (C-C motif) ligand 12 (CCL-12) (Supplementary Fig. S3A,B), significantly increased in BPA treated mice hearts. CCL2 receptor (CCR2), transcript levels were significantly increased at four weeks of BPA, even before the onset of clinical manifestation of heart disease, and remained elevated after 16 weeks of BPA **(**Fig. 2G**)**. Confocal analysis showed the colocalization of CCR2 with CD68 macrophages in the hearts of mice at eight weeks of administration of BPA **(**Supplementary Fig. 3C,D**)**. The accumulation of CCR2^+^ macrophages may represent a delay in polarization to M2 resolving macrophages^49^, and our data indeed indicate that M2 markers, including TFG-β and IL-10, were not increased even after 16 weeks of BPA (Fig. 2H,I).

### BPA induces myocardial hemorrhage and vascular leakage

**Figure 3:**
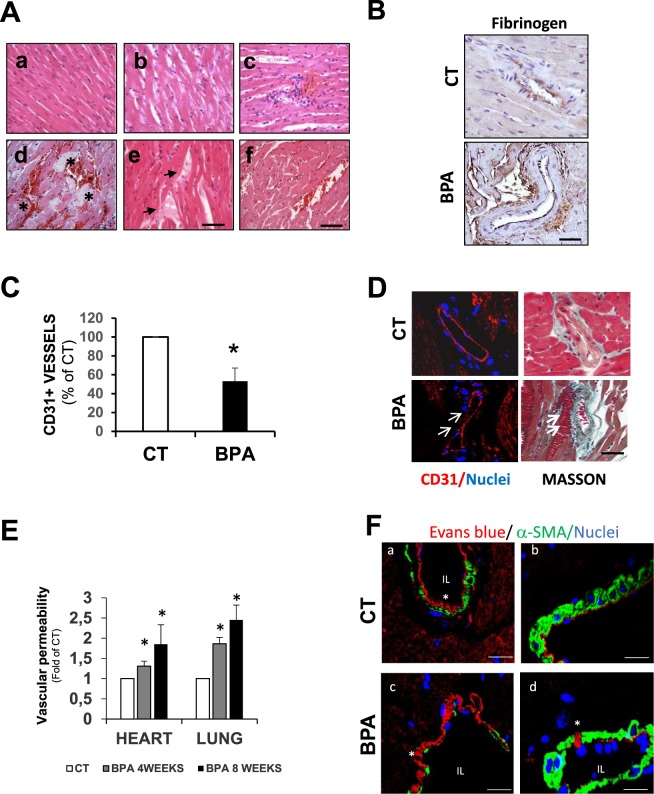
BPA induces hemorrhagic cardiac lesions, decreased vascularization, and vascular leakage. (**A**) Photomicrographs from hearts stained with hematoxylin/eosin of CT (a), BPA 4 weeks (b and c), BPA 8 weeks (d), and BPA 16 weeks (e and f). Asterisk mark extensive hemorrhagic areas with fibrin deposits. Arrows mark fibrin deposit between myocytes fibers (scale bar = 60 μm). (**B**) Immunohistochemistry of heart sections from CT and 8 weeks treated BPA mice stained for Fibrinogen. Scale bar = 25 μm (**C**) Blood vessel quantitation with an anti-CD31 endothelial cell marker in the heart of CT and 8 weeks BPA treated mice. CD31 + cells were labeled in red, and nuclei were labeled with Hoechst in blue. Three different heart regions were used to quantitate: RV, LV and IVS and at least 6 fields of each region were counted (n = 4 hearts per condition. Results are expressed as the number of CD31 + cells/mm^2^ and referred to CT). (**D**) *Left panel:* Representative confocal images from heart sections of CT and 8 weeks treated BPA mice showing immunostaining for CD31 (n = 4 mice per condition). *Right panel:* Masson trichrome staining of the same regions. Arrows mark hemorrhagic foci close to the blood vessel in BPA treated mice. Images are representative of n = 4 hearts per condition. (**E**) Vascular permeability in the heart and lungs measured 24 h after injection of Evans blue 2% expressed as ng dye/mg of tissue and referred to CT. The data are presented as mean ± SD, n = 6 mice per group. *p < 0.05 vs CT. F) Representative confocal images from heart sections of CT and 8 weeks treated BPA mice after injection with Evans blue (red fluorescence) followed by immunostaining for α-SMA (green). Nuclei were labeled with Hoechst in blue (n = 4 mice per condition). (a and b) CT hearts sections obtained at different magnifications showed no signs of EB extravasation. (c) and (d) are different sections of coronary arteries of BPA hearts with extravasation areas. Scale bar = 25 μm (a and c) and 50μm (b and d). IL = intraluminal area and (*) marks areas of EB extravasation.

Hematoxylin and eosin staining of heart sections from the BPA mice, exhibited a distinctive phenotype (Fig. 3A). Most of the hearts showed early signs of cardiac interstitial edema with significant disarranged myocardial fibers along with the presence of inflammatory cells (Fig. 3Ab). Interestingly, hemorrhage was apparent in 75 ± 2% of the hearts, as evidenced by the interstitial presence of red blood cells **(**Fig. 3Ac**)** with fibrin deposits **(**Fig. 3Ad), indicative of microvascular damage. Hemorrhagic lesions could be observed as early as four weeks after BPA administration. Large areas of hemorrhagic foci together with fibrin deposits were evidenced after 8 weeks of BPA treatment that were still present in the hearts of mice after 16 weeks, although the hemorrhagic lesions were not as extensive compared to 8 weeks **(**Fig. 3Ae,f**)**. A similar effect could also be observed in BPA treated mice at lower doses (4 × 10^−8^ and 4 × 10^−7^ M), which mimic environmental exposure **(**Supplementary Fig. 3A**)**. A positive fibrinogen staining near coronary arteries was observed, indicating plasma extravasation **(**Fig. 3B**)**. In addition, cardiac vascularization in BPA hearts was significantly reduced as detected by decreased number of CD31 positive cells **(**Fig. 3C**)**. In order to exclude BPA-induced coagulation defects, total platelet counts and coagulation tests (Prothrombin time (PT), Activated partial thromboplastin time (aPTT), Thrombin time(TT) and fibrinogen quantitation) were performed, but the results were similar for both groups CT and BPA. Hence, coagulation abnormalities could be excluded as the origin of cardiac hemorrhages **(**Supplementary Fig. 3B–E**)**.

To investigate whether BPA could promote structural changes in endothelial cells, we visualized CD31 positive endothelial cells within hemorrhagic areas by confocal microscopy. We observed gaps in the endothelial layer of injured vessels **(**Fig. 3D**)**, which suggest that BPA could modulate the endothelial barrier function. Next, we analyzed the permeability of endothelial cells by Evans blue (EB) dye extravasation, observing that EB extravasation increased by a 1,5-fold in hearst from 4 and 8 weeks-BPA treated mice compared to CT. Iinterestingly, in the lungs EB extravasation was increased by 3-fold compared to CT **(**Fig. 3E**)**. EB levels in livers and kidneys were similarly elevated in both BPA and CT mice due to fenestrated endothelium (not shown). EB extravasation in cardiac tissue was also observed by confocal microscopy (red fluorescence of EB), and coronary arteries were stained with α-smooth muscle actin antibody (α-SMA, green). Whilst EB staining was confined to he lumen of the coronary arteries in CT mice Fig. 3F(a,b), in BPA treated mice EB was detected in the outer wall of the coronary artery and intercalated between arterial muscle cells **(**Fig. 3Fc,d**)**.

Together, these results demonstrate that BPA increased vascular permeability and induced vascular leakage that could account for hemorrhagic damage that, together with a reduced vascularization, might lead to cardiac hypoperfusion and myocardial ischemia.

### BPA induced vascular injury is mediated by RIP3/CamKII dependent endothelial cell necroptosis

**Figure 4:**
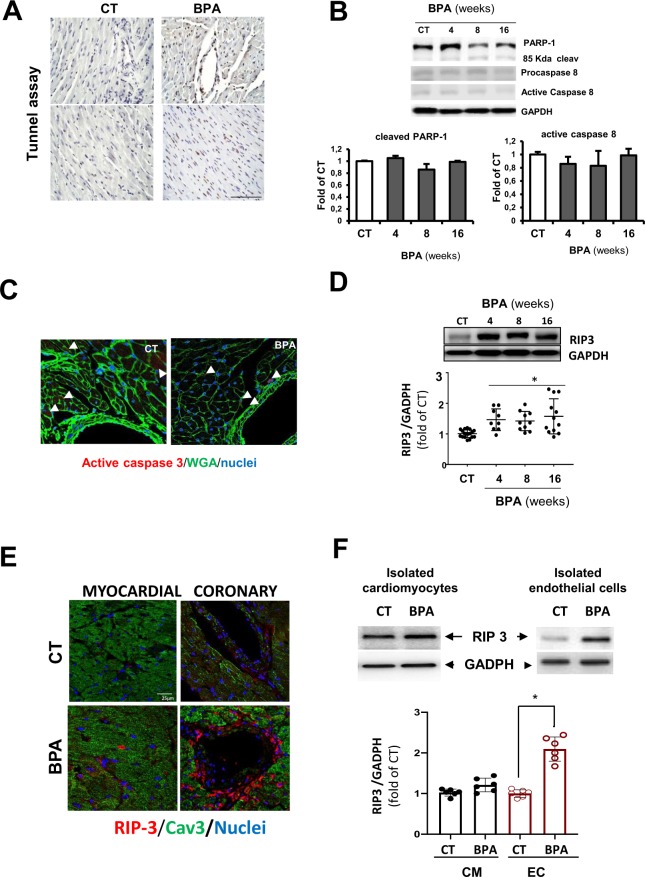
BPA induces cardiac necroptosis. (**A**) Detection of cell death by tunel assay in heart slides of CT and 8 weeks BPA treated mice. Brown staining indicates TUNEL-positive nuclei, and blue indicates living cells. A representative image of two regions of a heart sections is shown. Scale bar = 25 µm. Similar results were obtained in triplicate heart sections from n = 4 animals per condition. (**B**) Immunoblot detection of PARP-1, the 86 KDa cleaved form of PARP-1, procaspase, and 43 KDa active caspase 8 in total heart lysates from CT and BPA treated mice at 4, 8, and 16 weeks. GAPDH was used as a loading control. A representative immunoblot is shown. The densitometric analysis are shown below (data are shown as mean ± SD, n = 4 mice per condition). (**C**) Cardiac cross-sections were examined for activated caspase‐3 (red). WGA marked CM are green and Hoechst cell nuclei in blue. Representative images of findings in CT and 8 weeks treated BPA mice are shown (a total of 4 sections per heart were examined; n = 3 to 8 hearts per group. Scale bar = 50 μm). (**D**) RIP3 protein expression measured by western blotting in whole heart tissue from CT and BPA treated mice. GAPDH was used as a loading control. A representative immunoblot is shown. The densitometric analysis is shown below. Data shown represent each mouse cardiac RIP3 expression referred to CT (n = 8–12 mice per group, scale bar = 25 µm). (**E**) Representative confocal microscopy images of RIP3 (shown in red), in regions from CT and 8 weeks treated BPA mice. Scale bar = 25μm. The expression of Caveolin-3 was used as a cardiac myocyte cell marker (Cav3, in green). Nuclei were stained with Hoechst (blue). Similar results were obtained in n = 4 hearts per condition. (**F**) RIP3 protein expression measured by western blotting in freshly isolated CM or EC from CT and 8 weeks BPA treated mice. The bar graph shows mean ± SD of RIP3/GADPH average refered to CT (n = 6 hearts per condition, *p < 0.05 vs CT in ECs).

Inflammation and cell death are associated with the development of heart disease, especially under increased oxidative stress conditions, as observed in BPA treated mice^50,51^. To identify the mechanisms underlying vascular leakage and myocardial hemorrhages induced by BPA, we performed tunel assay in hearts from 8 weeks BPA treated mice, observing increased cell death, especially at the coronary endothelium and within the vascular wall, compared to CT **(**Fig. 4A**)**. There was a 2.8 ± 1.06 fold increase in total tunel positive cells in heart slides at 8 weeks after BPA treatment, while in areas containing coronary vessels, the increase is 9,89 ± 0.856 indicating a higher susceptibility of vascular cells to BPA induced cell death.

Western blot analysis of whole heart lysate showed neither changes in caspase 3 activity, as reflected by the Poly [ADP-ribose] polymerase 1 (PARP-1) cleavage, nor increased caspase 8 activity **(**Fig. 4B**)**. Similarly, confocal microscopy analysis of CT and 8 weeks BPA heart sections revealed few differences in active caspase 3 levels **(**Fig. 4C**)**, suggesting that an apoptosis-independent mechanism is involved in BPA-induced endothelial cell death.

To confirm that endothelial cells were not undergoing apoptotic cell death, we treated mouse aortic endothelial cells (MAEC) with 0-10^−4^ M BPA for 24 h. BPA reduced cell viability from 10^−7^ M **(**Supplementary Fig. S5A**)**. Therefore MAEC were treated with 10^−6^ M of BPA for 24, 48 and 72 h and the level of caspase 3 activity, as detected by PARP-1 cleavage, and as well caspase 8 were studied by western blot. We found no caspase activation in BPA treated cells compared to vehicle treated cells (CT) **(**Supplementary Fig. S5B**)**. However, the treatment of cardiomyocyte cells (H9c2) with different concentrations of BPA during 24 h, did no reduce cell viability except at the highest concentration (10^−4^ M). **(**Supplementary Fig. S5C**)**. Together, these results confirmed that BPA targets endothelial cells by an apoptotic independent form of cell death.

Interestingly, caspase-8 induces apoptosis but also serves as a significant regulator of necroptosis. Active caspase 8 can cleave RIP1 and 3, thus preventing necroptotic signaling^52,53^.To explore if necroptosis contributes to BPA induced coronary vessel injury, we investigated RIP3 levels in whole cardiac tissue of BPA treated mice. RIP 3 protein expression, which is critical to initiate necroptosis, was increased in the myocardium of BPA treated animals for 4,8 weeks and 16 weeks **(**Fig. 4D**)**. Confocal microscopy revealed intense labeling of RIP 3 after 8 weeks of treatment with BPA at the endothelial lining of coronary vessels and around blood vessels. However, RIP3 staining in cardiomyocytes was slightly more intense that in CT hearts **(**Fig. 4E**)**.

To clarify which cardiac cells were contributing to increased RIP 3 expression, we isolated cardiomyocytes (CM) and non-myocyte cellular fractions from CT and 8 weeks-BPA treated mice. Non-cardiomyocyte cells consisted mostly in endothelial cells, as detected by positive CD31 staining and the absence of α-SMA staining. Although CM from CT and 8 weeks BPA treated mice showed no increase in RIP 3 levels, the endothelial cell fraction showed significant increased RIP 3 expression **(**Fig. 4F**)**. Similarly, western blot analysis of RIP3 expression on H9c2 cells showed no differences between CT and BPA treated cells **(**Supplementary Fig. S5D**)**. Together, those results indicate that BPA may initiate a RIP 3-necroptotic pathway in vascular cells.

**Figure 5:**
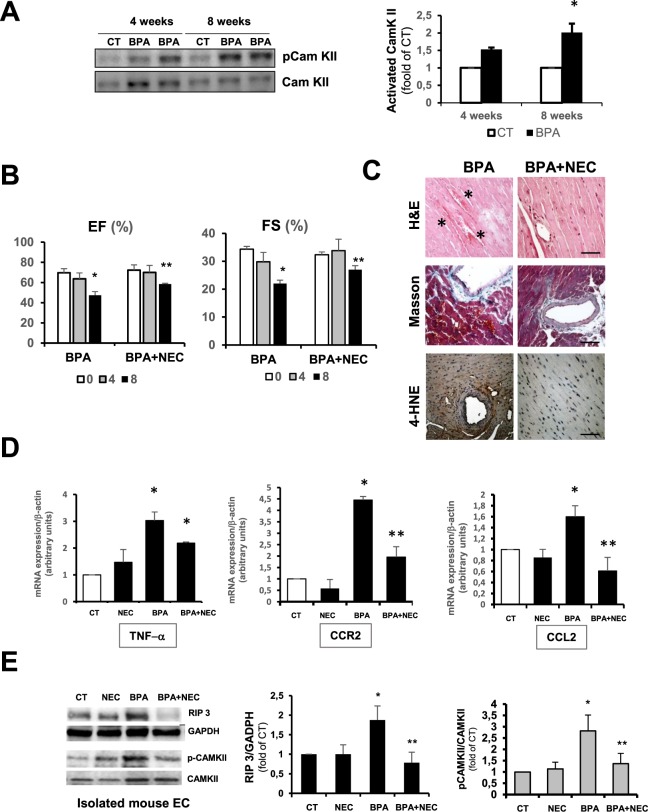
Necroptosis inhibition with necrostatin-1 reduces the adverse cardiac effects of BPA. (**A**) *Left panel:* representative immunoblot showing phosphorylated-CamKII and total CamKII protein expression in whole heart extracts from CT and BPA treated mice at 4 and 8 weeks. *Right panel:* densitometric analysis shown as mean ± SD (n = 4; *p < 0.05 vs control CT). Mice were treated with BPA and BPA and necrostatin-1 (NEC) for 4 and 8 weeks. (**B**) Echocardiography was performed at indicated time points for LV Ejection fraction (EF) and fractional shortening (FS) measurement (BPA n = 8 and BPA + NEC, n = 6 mice per group; *p < 0.05 vs. time 0, **p < 0.05 vs. BPA 8 weeks). (**C**) Representative photomicrographs from hearts stained with hematoxylin/eosin to visualize hemorrhagic lesions, Masson´s trichrome to detect fibrosis, and 4-hydroxinonenal as a marker of oxidative stress (n = 6 animals per group). Asterisk mark extensive hemorrhagic areas. Scale bar = 60 μm. (**D**) RT-qPCR of CT, NEC, BPA and BPA + NEC 8 weeks treated mice showing cardiac mRNA expression of TNF-α, CCL2 and CCR2 (n = 4 per condition with triplicates in each determination, *p < 0.05 vs. CT; **p < 0.01 vs. BPA). (**E**) Immunoblot analysis of RIP 3, phosphorylated-CamKII and total CamKII protein expression in protein extracts from freshly isolated ECs from CT, NEC, BPA, and BPA + NEC treated mice at 8 weeks. A representative immunoblot is shown and densitometric analysis shown as mean ± SD (n = 6; *p < 0.05 vs control CT; **p < 0.05 vs BPA).

Recently it has been described that necroptosis can proceed in a RIP 1 independent pathway, activating Ca^2+^-CamKII to regulate mitochondrial permeability transition pore (mPTP) opening^54,55^. The opening of the mPTP in response to ischemia-reperfusion injury has been linked to cell death in the heart and other organs^56,57^. Here we found that BPA increased CamKII phosphorylation in whole heart homogenates at 4 and 8 weeks of BPA treatment **(**Fig. 5A**)**.

To better comprehend the relationship between BPA and cardiac necroptosis, we treated mice with BPA and the necroptosis inhibitor Necrostatin-1(NEC-1) for 4 and 8 weeks. Functional analysis revealed that necrostatin was able to partially revert the impairment in cardiac contractility caused by BPA **(**Fig. 5B**)**. Histological analysis also evidenced a less pronounced cardiac injury compared with BPA treated mice with less oxidative stress as detected by 4-HNE staining (Fig. 5C, lower panels). More importantly, we found smaller hemorrhagic lesions (Fig. 5C H&E upper, and Masson Trichrome middle panels). NEC-1 treatment also produced a reduced inflammatory response compared to BPA alone, as detected by the expression of pro-inflammatory mRNA corresponding to TNF-α, CCR2 and CCL2 (Fig. 5D). RIP 3 levels were studied in isolated EC protein samples, finding a reduction of RIP 3 expression in NEC-1 + BPA compared to BPA alone, which correlates with reduced p-CamKII expression in endothelial cells, indicating a link between BPA and induced endothelial RIP 3, CamKII activation, and cardiac injury **(**Fig. 5E**)**.

### BPA induced endothelial necroptosis is mediated by RIP3/CamKII pathway

**Figure 6:**
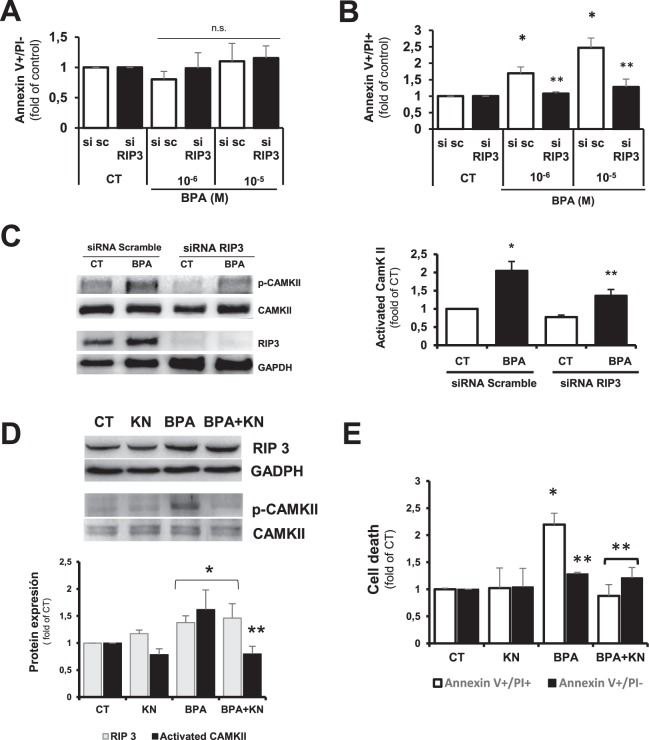
BPA induces endothelial cell necroptosis via RIP3/CamKII. (**A**,**B**) Flow cytometry analysis of MAEC transfected with RIP3-specific siRNA (si RIP3) or non-silencing siRNA scramble (si sc) and treated with BPA at 10^−6^ M and 10^−5^ M for 24 h. Cells were stained with annexin V-FITC and propidium iodide. Data are shown as mean ± SD (n = 4 per condition with duplicates in each determination). (**A**) apoptotic cells annexin V^+^/IP^−^ (ns; non significant) and (**B**) annexin V^+^/PI^+^ cells. (*p < 0.05 vs. CT si sc, **p < 0.05 vs. BPA). (**C**) Immunoblot analysis of phosphorylated-CamKII, total CamKII, RIP3, and GADPH protein expression in CT and BPA (10^−6^ M) treated MAEC transfected as above. A representative experiment is shown (n = 4). Data are expressed as mean ± SD and referred to CT, *p < 0.05 versus CT siRNA-scramble MAEC; **p < 0.05 vs CT SiRNA RIP3. (**D**) Immunoblot analysis of RIP 3 and phosphorylated CamKII and total CamKII protein expression in protein extracts from MAEC treated with vehicle (CT), 10^−6^ M BPA (BPA), KN-93 (inhibitor of CamKII, 10^−6^ M) and BPA + KN for 24 h. A representative experiment is shown (n = 4). Data are presented as mean ± SD and refered to CT; *p < 0.05 vs. CT;**p < 0.05 vs. BPA. (**E**) Flow cytometry analysis of MAEC treated with vehicle (CT), BPA, KN-93, and BPA + KN for 24 h. Cells were stained with annexin V-FITC and propidium iodide. Data are shown as mean ± SD and referred to CT (n = 4 with duplicates in each determination,*p < 0.05 vs. CT or KN and **p < 0.05 vs. BPA AnexinV^+^PI^+^).

We explored the implication of CamKII on endothelial cell necroptosis, using cultured mouse aortic endothelial cells (MAEC). BPA increased the expression of RIP 3 (Supplementary Fig. 5E), similarly to the response induced by BPA in the cardiac endothelial cell fraction. To test whether RIP 3 mediates BPA-induced necroptosis, RIP 3 expression was silenced by siRNA and cell death assayed by flow cytometry. Administration of BPA in RIP3-silenced MAEC had no effect on the number of apoptotic cells (annexin V+/PI−), but necroptosis (annexinV+/PI+) was significantly reduced (Fig. 6A,B and Supplementary Fig. 5F), as well as the BPA-induced activation of CamKII (Fig. 6C). Pharmacological inhibition of CamKII activity with Kn-93 (KN) did not affect BPA induced RIP3 expression (Fig. 6D) but was able to decrease BPA induced necroptosis (Fig. 6E and Supplementary Fig. 6G). Thus, BPA stimulation of endothelial necroptosis proceeds via RIP 3 induction of CamKII signaling.

Taken together, these results suggest that BPA induces necroptosis in endothelial cells, contributing to coronary damage, vascular leakage, and chronic inflammation leading ultimately to myocardial injury.

## Discussion

In this study, we investigated the mechanism of BPA-induced damage in male mice hearts. Our results demonstrate that chronic administration of BPA causes hypertension and cardiac hypertrophy, leading to impaired cardiac function and cardiac hemorrhage. We have uncovered a novel pathogenic role of BPA to induce coronary endothelial cell death by necroptosis through the RIP3-CamKII pathway causing vascular leakage leading to cardiac hypoperfusion.

In our mouse model, BPA induced heart dysfunction with reduced EF, FS, increased IVS thickness, abnormalities on cardiac electric conductivity, hypertension, and cardiac congestion. These results are in agreement with several reports describing a pro-arrhythmogenic effect of BPA in female rat hearts^9,58^ and impaired cardiac function and contractility through alterations of myocyte calcium handling initiated by estrogen receptor^15^. Although only male mice were used in our study, an effect mediated by the estrogen receptor cannot be excluded entirely. Nonetheless, our results show that BPA slowed electrical conduction in male hearts with first degree AV block, which is in agreement with BPA effects reported by Posnack NG *et al*. in rat hearts^59,60^. In addition, life long administration of BPA in doses similar to human exposure also demonstrated an arrhythmogenic effect with evidence of cardiomyopathy^61^. This effect is relevant to human health since slowed conduction could represent a mechanism of reentrant arrhythmias, which can also cause tachycardia^62^ and could explain the increased heart rate observed in our mice.

Interestingly, the effect of BPA on electric conductivity has been found particularly pronounced under pathophysiological conditions such as stress or ischemic injury^9,10^. Indeed, dobutamine stress tests revealed that chronotropic and inotropic responses were impaired, and the coronary reserve seemed compromised in BPA treated mice. Since BPA may cause vasoconstriction due to a decrease in nitric oxide (NO) production and therefore decreased blood supply to the heart, ischemia could account for the arrhythmogenic behavior found in our study^23^. Besides, BPA treated mice might have a defect on the adrenergic signaling pathway, which could compromise Ca^2+^ homeostasis or in G-protein–coupled receptor signaling^7^. Moreover, BPA treated mice exhibit increased expression and activity of CamKII, which is also increased in animal models of heart failure and in humans in failing or arrhythmia-prone myocardium, pointing to a possible mechanism to explain BPA induced cardiac contractile dysfunction and arrhythmias^55^.

Our studies detected the infiltration of macrophages into the heart early after BPA treatment, accompanied by an increased expression of inflammatory cytokines CCL2, TNF-α, CCR2, CCL7 and CCL-12, markers of inflammatory M1 macrophages even at 16 weeks after exposure to BPA. CCR2^+^ macrophages could be detected at 8 weeks. In animal models of pressure overload, CCR2^+^ macrophages play a critical role leading to subsequent cardiac T-cell expansion, pathological LV remodeling, and late heart failure^63^. The presence of CCR2^+^ macrophages, together with reduced myocardial vascularization, may be indicative of defective reparative processes. In agreement with our results, chronic administration of BPA or its substitute, Bisphenol S (BPS), can reduce active cardiac remodeling in response to myocardial infarction, increasing metalloproteinase (MMP) expression in male mice^12^. Moreover, the exposure of resting monocytes to BPA increased polarization to activated macrophages^64^. It has also been reported that positive CCR2 macrophages enriched in the AV node may cause abnormal atrioventricular electrical conduction^65^. Thus, recruitment of CCR2^+^ pro-inflammatory macrophages may also contribute to the arrhythmogenic AV block in response to chronic exposure to BPA.

Most studies about the cardiac effect of BPA focused on electrical conduction and structural or metabolic abnormalities on cardiomyocytes, however, data on its effects on cardiac endothelial cells are still missing. Interestingly, BPA cardiac effects presented accompanied by myocardial hemorrhage. Gear *et al*., described that female rats exposed to higher doses of BPA for six months developed a cardiomyopathy with hemosiderin containing macrophages, indicative of previous microvasculature damage and hemorrhage^11^. However, the potential causes of BPA induced hemorrhages had not been investigated so far. Our first hypothesis was that BPA targeted the coagulation cascade, but none of the coagulation parameters were altered. Therefore,we speculated that hemorrhages could be secondary to ischemia and hypertension as cardiac hemorrhagic lesions have been described in response to ischemic reperfusion^66^. Here, we observed BPA-induced hemorrhagic foci already at 4 weeks, and although no ischemic events were detected in the ECG in resting conditions, after dobutamine challenge, 70% of the mice showed ST segments depression, indicative of cardiac ischemia. Furthermore, heart rate increased in BPA treated mice and some animals presented even further increases in HR after 8 weeks of BPA treatment. These conditions can contribute to increased oxidative stress, as detected by increased lipid peroxidation, and accumulation of oxidative insults could lead to endothelial wall injury mimicking the conditions present during ischemia-reperfusion. Accordingly, Aboul Ezz *et al*. reported increased lipid peroxidation, decreased glutathione (GSH) levels and catalase activity after 6 to 10-week BPA administration^67^. We also found decreased blood vessel density in BPA treated-mice, which, together with the increased cardiomyocyte mass, could also reduce myocardial oxygen supply leading to ischemia.

The increased Evans Blue extravasation in the hearts and lungs of BPA treated mice support the hypothesis that BPA increases vascular permeability contributing to heart damage. Indeed, the extensive hemorrhagic areas observed at eight weeks also indicate vascular fragility and rupture of coronary microvessels. We have demonstrated that BPA decreases endothelial NO and increases oxidative stress leading to endothelial dysfunction and hypertension^23^. Since BPA can cause hypertension in humans^8^ and mice^23^, another cause for the hemorrhages may be related to hypertensive degenerative or inflammatory changes affecting small arterioles, which could cause structural weakening and eventually vascular wall rupture. Studies on mouse models of hypertension-induced cerebral microvascular hemorrhages (CMH) demonstrate that pathogenesis of the CMHs involves a weakening of the vessel wall by upregulation of MMPs or oxidative stress-dependent activation of MMPs, such as MMP2 and MMP9^68^. Multiple lines of evidence suggest that NO regulates activation or expression of MMPs, and that decreased NO bioavailability promotes MMP activation in the vascular wall^69,70^. In the present study, we showed that hypertension increases at 16 weeks. Although MMPs levels were not examined here, others have recently described in a model of lifelong exposure to BPA a decreased recovery after myocardial infarction associated with adverse cardiac remodeling due to increased MMP2 and MMP9 expression^12^. Besides, Belcher *et al*. reported that cardiac transcriptome after BPA exposure caused sex-specific dysregulation of the collagen extracellular matrix (ECM)^7^. Since BPA impairs NO production due to eNOS uncoupling, BPA could likely affect ECM turnover via NO signaling leading to a weakening of coronary vascular wall, which in addition to ischemia, could be the culprit behind microhemorrhages. A detailed characterization of the molecular mechanism responsible for early microvascular hemorrhage is critical to understand the cardiac effects of BPA, especially during cardiac ischemia, since myocardial hemorrhage has been associated with human adverse remodeling and adverse health outcome in the longer term^71^.

Here, we provide a potential mechanism for detrimental cardiac effects of BPA *in vivo*, finding the activation of necroptosis cascade as the cause of endothelial cell death leading to hypoperfusion and ischemia. Our data showed increased cell death in hearts from BPA treated mice, especially in the areas containing blood vessels, in the absence of increased apoptotic markers. Necroptosis is a newly discovered pathway of programmed cell death with an essential role in human ischemic injury^72^. RIP 3 is a critical determinant in the necroptotic pathway and is expressed in the myocardium^73^. The process of necroptosis is dependent on the activation of the RIP 1–RIP 3–MLKL axis initiated by inflammatory TNF-α and other pro-apoptotic stimuli such as oxidative stress^74^. Our results establish a role for RIP 3 in BPA-induced endothelial cell injury since, since RIP 3 levels increased only in endothelial cells and perivascular areas while this protein was not found increased in cardiomyocytes. Nonetheless, we can not exclude a BPA role in RIP 3 mediated necroptosis in fibroblast or macrophages. The necroptotic pathway is also proven to relate to vascular disease as elevated RIP 3 levels are detected in ischemic stroke, atherosclerosis, and aortic aneurisms^74^. Interestingly, BPA decreased endothelial cell viability in a dose-dependent manner but failed to induce apoptotic cell death. In our study, gene silencing of RIP 3 reversed the effect of BPA on endothelial cell necroptosis. It has been reported that RIP 3 knockout decreases myocardial necroptosis in myocardial infarction^73^. Accordingly, the treatment of mice with BPA and the necroptosis inhibitor NEC-1 reversed cardiotoxic effects of BPA producing fewer hemorrhages and less inflammation. In support of our results, Chen J. *et al*. showed that NEC-1 prevented the disruption of the brain-blood barrier reducing brain hemorrhages^75^. However, NEC-1 only induced a moderate improvement of cardiac function so the possibility of a direct effect of BPA on myocardium by a different pathway cannot be completely ruled out.

Two mechanisms can contribute to BPA triggering the necroptotic pathway: early inflammation, which increased TNF-α, a known initiator of RIP 1/3 necroptosis, and, secondly, inhibition of caspase-8 activity which allowed RIP 3 phosphorylation and activation. Recent reports documented that RIP3 can also facilitate inflammation independent of myocardial necrosis by releasing damage-associated molecular patterns (DAMPS)^76^. In our study, inflammation appears as early as 4 weeks and remains elevated after 16 weeks. Thus, RIP 3-induced necroptosis could be initiated by TNF-α and oxidative stress, and later could exacerbate the inflammatory conditions induced by BPA in later stages. The necroptosis-inflammatory response might explain the lack of M2 phenotype macrophages found at 16 weeks. This hypothesis is supported by the decreased inflammation found in BPA + NEC-1-treated mouse.

BPA-dependent cardiac damage could be initiated by inflammation and oxidative stress increasing CamKII activation. Moreover, oxidation of CamKII might be the primary mechanism for myocardial rupture following myocardial infarction^77^. Here we have shown increased activation of CamKII in hearts from 4 and 8 weeks BPA treated mice. CamKII activation by autophosphorylation and oxidation has been proposed as a potential mechanism to activate necroptosis and conversely CamKII has been proposed as an alternative substrate of RIP 3, leading to myocardial necroptosis during ischemia-reperfusion injury^28^. Our data demonstrate that inhibition of CamKII activity with KN-93 decreases necroptosis but does not affect BPA increased-RIP 3. However, RIP 3 silencing prevented CamKII phosphorylation and necroptosis induction. Therefore our results point to RIP 3 acting through CamKII to induce necroptosis in response to BPA.

**Figure 7:**
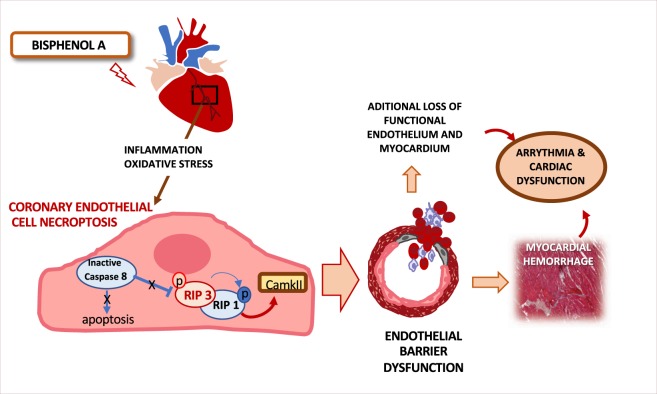
Mechanism of the cardiotoxic effects of BPA. BPA induces early inflammation and oxidative stress leading to endothelial cell death. BPA activates the RIP 3-CamKII necroptotic pathway at endothelial cells. Necroptosis could be contributing to exacerbate the inflammatory conditions induced by BPA although direct effects on myocardium can not be excluded. Decreased endothelial barrier function and weakening of the coronary vascular wall in the setting of hypertension may cause ventricular hemorrhages, cardiac and lung congestion, ultimately leading to heart failure.

In summary, our study provides a mechanism to explain the cardiotoxic effects of BPA. BPA activates the RIP 3-CamKII necroptotic pathway leading to endothelial cell death. Decreased endothelial barrier function and weakening of the coronary vascular wall in the setting of hypertension may cause ventricular hemorrhages, cardiac, and lung congestion, which ultimately led to heart failure (summarized in Fig. 7). Targeting the RIP 3 pathway may have beneficial effects in BPA induced cardiac damage, mainly when associated with ischemia.

Our results are particularly interesting because exposure to this common environmental chemical could pose an additional risk for individuals with preexisting cardiac conduction abnormalities, cardiac disease, and other cardiovascular risk factors.

## Supplementary information

Supplementary information.

## Data Availability

All data generated or analyzed during this study are included in this published article and the Supplementary Information files

## References

[1] Krishnan AV, Stathis P, Permuth SF, Tokes L, Feldman D (1993). Bisphenol-A: an estrogenic substance is released from polycarbonate flasks during autoclaving. Endocrinology.

[2] Kuiper GG (1997). Comparison of the ligand binding specificity and transcript tissue distribution of estrogen receptors alpha and beta. Endocrinology.

[3] Wang YX (2019). Urinary levels of bisphenol A, F and S and markers of oxidative stress among healthy adult men: Variability and association analysis. Environ. Int..

[4] Calafat AM, Ye X, Wong L-Y, Reidy JA, Needham LL (2008). Exposure of the U.S. population to bisphenol A and 4-tertiary-octylphenol: 2003-2004. Environ. Health Perspect..

[5] Provvisiero D (2016). Influence of Bisphenol A on Type 2 Diabetes Mellitus. Int. J. Environ. Res. Public Health.

[6] Xiong Q (2015). Elevated Serum Bisphenol A Level in Patients with Dilated Cardiomyopathy. Int. J. Environ. Res. Public Heal..

[7] Belcher SM, Gear RB, Kendig EL (2015). Bisphenol a alters autonomic tone and extracellular matrix structure and induces sex-specific effects on cardiovascular function in male and female CD-1 mice. Endocrinology.

[8] Bae S, Hong YC (2015). Exposure to bisphenol a from drinking canned beverages increases blood pressure: Randomized crossover trial. Hypertension.

[9] Yan Sujuan, Chen Yamei, Dong Min, Song Weizhong, Belcher Scott M., Wang Hong-Sheng (2011). Bisphenol A and 17β-Estradiol Promote Arrhythmia in the Female Heart via Alteration of Calcium Handling. PLoS ONE.

[10] Yan S (2013). Low-dose bisphenol A and estrogen increase ventricular arrhythmias following ischemia–reperfusion in female rat hearts. Food Chem. Toxicol..

[11] Gear R, Kendziorski JA, Belcher SM (2017). Graduate Training Program, P. & Lett Author manuscript, T. Effects of bisphenol A on incidence and severity of cardiac lesions in the NCTR-Sprague-Dawley rat: A CLARITY-BPA study. Toxicol. Lett..

[12] Kasneci A (2017). From the Cover: Lifelong Exposure of C57bl/6n Male Mice to Bisphenol A or Bisphenol S Reduces Recovery From a Myocardial Infarction. Toxicol. Sci..

[13] Shang J (2019). Recovery From a Myocardial Infarction Is Impaired in Male C57bl/6 N Mice Acutely Exposed to the Bisphenols and Phthalates That Escape From Medical Devices Used in Cardiac Surgery. Toxicol. Sci..

[14] Molina-Molina JM (2019). Determination of bisphenol A and bisphenol S concentrations and assessment of estrogen- and anti-androgen-like activities in thermal paper receipts from Brazil, France, and Spain. Environ. Res..

[15] Ramadan M (2018). Disruption of neonatal cardiomyocyte physiology following exposure to bisphenol-a. Sci. Rep..

[16] Gao X, Ma J, Chen Y, Wang HS (2015). Rapid responses and mechanism of action for low-dose bisphenol S on *ex vivo* rat hearts and isolated myocytes: Evidence of female-specific proarrhythmic effects. Environ. Health Perspect..

[17] Jiang Y (2015). BPA-induced DNA hypermethylation of the master mitochondrial gene PGC-1α contributes to cardiomyopathy in male rats. Toxicology.

[18] Ferguson, M., Lorenzen-Schmidt, I. & Pyle, W. G. Bisphenol S rapidly depresses heart function through estrogen receptor-β and decreases phospholamban phosphorylation in a sex-dependent manner. *Sci. Rep*. **9** (2019). 10.1038/s41598-019-52350-y PMC6828810 31685870

[19] Patel BB, Di Iorio M, Chalifour LE (2014). Metabolic response to chronic bisphenol A exposure in C57bl/6n mice. Toxicol. Reports.

[20] Sui Y, Park SH, Wang F, Zhou C (2018). Perinatal Bisphenol A Exposure Increases Atherosclerosis in Adult Male PXR-Humanized Mice. Endocrinology.

[21] LaKind, J. S., Goodman, M. & Naiman, D. Q. Use of NHANES Data to Link Chemical Exposures to Chronic Diseases: A Cautionary Tale. *PLoS One* **7** (2012). 10.1371/journal.pone.0051086 PMC3515548 23227235

[22] Mao N, Gao Q, Hu H, Zhu T, Hao L (2019). BPA disrupts the cardioprotection by 17β-oestradiol against ischemia/reperfusion injury in isolated guinea pig hearts. Steroids.

[23] Saura M (2014). Oral administration of bisphenol A induces high blood pressure through angiotensin II/CaMKII-dependent uncoupling of eNOS. FASEB J..

[24] Dominic Swaminathan P, Purohit A, Hund TJ, Anderson ME (2012). Review Calmodulin-Dependent Protein Kinase II: Linking Heart Failure and Arrhythmias. Circ Res.

[25] Anderson ME (2005). Calmodulin kinase signaling in heart: An intriguing candidate target for therapy of myocardial dysfunction and arrhythmias. Pharmacology and Therapeutics.

[26] Yang Y (2006). Calmodulin kinase II inhibition protects against myocardial cell apoptosis *in vivo*. Am. J. Physiol. Circ. Physiol..

[27] Feng N, Anderson ME (2017). CaMKII is a nodal signal for multiple programmed cell death pathways in heart. J. Mol. Cell. Cardiol..

[28] Zhang T (2016). CaMKII is a RIP3 substrate mediating ischemia- and oxidative stress-induced myocardial necroptosis. Nat. Med..

[29] Gao X, Wang HS (2014). Impact of bisphenol A on the cardiovascular system - Epidemiological and experimental evidence and molecular mechanisms. Int. J. Environ. Res. Public Health.

[30] Takahashi, N. *et al*. Necrostatin-1 analogues: Critical issues on the specificity, activity and *in vivo* use in experimental disease models. *Cell Death Dis*. **3** (2012). 10.1038/cddis.2012.176 PMC3542611 23190609

[31] Lopez-Rivera E (2005). Matrix metalloproteinase 13 mediates nitric oxide activation of endothelial cell migration. Proc. Natl. Acad. Sci..

[32] Wang, J. M., Chen, A. F. & Zhang, K. Isolation and primary culture of mouse aortic endothelial cells. *J. Vis. Exp*. **2016** (2016). 10.3791/52965 PMC5226426 28060318

[33] Watkins SJ, Borthwick GM, Arthur HM (2011). The H9C2 cell line and primary neonatal cardiomyocyte cells show similar hypertrophic responses *in vitro*. Vitr. Cell. Dev. Biol. - Anim..

[34] Ackers-Johnson M (2016). A Simplified, Langendorff-Free Method for Concomitant Isolation of Viable Cardiac Myocytes and Nonmyocytes From the Adult Mouse Heart. Circ. Res..

[35] Wehrens X (2000). Mouse electrocardiography An interval of thirty years. Cardiovasc. Res..

[36] Cuadrado I (2016). EMMPRIN-Targeted magnetic nanoparticles for *in vivo* visualization and regression of acute myocardial infarction. Theranostics.

[37] Gao, S., Ho, D., Vatner, D. E. & Vatner, S. F. Echocardiography in Mice. in *Current Protocols in Mouse Biology*, 10.1002/9780470942390.mo100130 (John Wiley & Sons, Inc., 2011). 10.1002/9780470942390.mo100130 PMC3130310 21743841

[38] Radu, M. & Chernoff, J. An *in vivo* assay to test blood vessel permeability. *J. Vis. Exp*., 10.3791/50062 (2013). 10.3791/50062 PMC3639515 23524912

[39] Wick, M. J., Harral, J. W., Loomis, Z. L. & Dempsey, E. C. An optimized evans blue protocol to assess vascular leak in the mouse. *J. Vis. Exp*. **2018** (2018). 10.3791/57037 PMC6235160 30272649

[40] Wang HL, Lai TW (2014). Optimization of Evans blue quantitation in limited rat tissue samples. Sci. Rep..

[41] Herranz B (2012). Integrin-Linked Kinase Regulates Vasomotor Function by Preventing Endothelial Nitric Oxide Synthase Uncoupling. Circ. Res..

[42] Ramos-Vara JA (2005). Technical aspects of immunohistochemistry. Veterinary Pathology.

[43] Makino A, Platoshyn O, Suarez J, Yuan JXJ, Dillmann WH (2008). Downregulation of connexin40 is associated with coronary endothelial cell dysfunction in streptozotocin-induced diabetic mice. Am. J. Physiol. Physiol..

[44] Schindelin J (2012). Fiji: an open-source platform for biological-image analysis. Nat. Methods.

[45] Butler RE (2017). Susceptibility of Mycobacterium tuberculosis -infected host cells to phospho-MLKL driven necroptosis is dependent on cell type and presence of TNFα. Virulence.

[46] van Meerloo Johan, Kaspers Gertjan J. L., Cloos Jacqueline (2011). Cell Sensitivity Assays: The MTT Assay. Methods in Molecular Biology.

[47] Bologna-Molina R, Damián-Matsumura P, Molina-Frechero N (2011). An easy cell counting method for immunohistochemistry that does not use an image analysis program. Histopathology.

[48] Suetomi T, Miyamoto S, Brown JH (2019). Inflammation in nonischemic heart disease: Initiation by cardiomyocyte CaMKII and NLRP3 inflammasome signaling. American Journal of Physiology - Heart and Circulatory Physiology.

[49] Bajpai G (2018). The human heart contains distinct macrophage subsets with divergent origins and functions. Nat. Med..

[50] Kania G, Blyszczuk P, Eriksson U (2009). Mechanisms of Cardiac Fibrosis in Inflammatory Heart Disease. Trends Cardiovasc. Med..

[51] Marín-García J (2016). Cell death in the pathogenesis and progression of heart failure. Heart Fail. Rev..

[52] Kaiser WJ (2011). RIP3 mediates the embryonic lethality of caspase-8-deficient mice. Nature.

[53] Oberst A (2011). Catalytic activity of the caspase-8–FLIPL complex inhibits RIPK3-dependent necrosis. Nature.

[54] Yang Z (2018). Melatonin attenuates chronic pain related myocardial ischemic susceptibility through inhibiting RIP3-MLKL/CaMKII dependent necroptosis. J. Mol. Cell. Cardiol..

[55] Zhang T, Brown JH (2004). Role of Ca2+/calmodulin-dependent protein kinase II in cardiac hypertrophy and heart failure. Cardiovasc. Res..

[56] Joiner M-LA (2012). CaMKII determines mitochondrial stress responses in heart. Nature.

[57] Morciano G (2017). Mechanistic Role of mPTP in Ischemia-Reperfusion Injury. Adv. Exp. Med. Biol..

[58] Gao X, Liang Q, Chen Y, Wang H-S (2013). Molecular mechanisms underlying the rapid arrhythmogenic action of bisphenol A in female rat hearts. Endocrinology.

[59] Posnack NG (2015). Physiological response of cardiac tissue to bisphenol a: alterations in ventricular pressure and contractility. Am. J. Physiol. Circ. Physiol..

[60] Posnack NG (2014). Bisphenol A exposure and cardiac electrical conduction in excised rat hearts. Environ. Health Perspect..

[61] NIEHS (2018). The CLARITY-BPA Core Study: A Perinatal and Chronic Extended-Dose-Range Study of Bisphenol A in Rats. Ntp Rr.

[62] Roden DM (1996). Ionic mechanisms for prolongation of refractoriness and their proarrhythmic and antiarrhythmic correlates. Am. J. Cardiol..

[63] Patel B (2018). CCR2+Monocyte-Derived Infiltrating Macrophages Are Required for Adverse Cardiac Remodeling During Pressure Overload. JACC Basic to Transl. Sci..

[64] Patel BB (2015). Chronic Exposure to Bisphenol A Reduces Successful Cardiac Remodeling After an Experimental Myocardial Infarction in Male C57bl/6n Mice. Toxicol. Sci..

[65] Hulsmans M (2017). Macrophages Facilitate Electrical Conduction in the Heart. Cell.

[66] Wong C-K (2015). Intra-myocardial hemorrhage in STEMI reperfusion: An alternative explanation for failures from “augmented” fibrinolysis regimes and fibrinolysis-facilitated PCI?. Int. J. Cardiol..

[67] Aboul Ezz HS, Khadrawy YA, Mourad IM (2013). The effect of bisphenol A on some oxidative stress parameters and acetylcholinesterase activity in the heart of male albino rats. Cytotechnology.

[68] Ungvari Z, Tarantini S, Kirkpatrick AC, Csiszar A, Prodan CI (2017). Cerebral microhemorrhages: mechanisms, consequences, and prevention. Am. J. Physiol. - Hear. Circ. Physiol..

[69] Dey NB, Lincoln TM (2012). Possible involvement of Cyclic-GMP-dependent protein kinase on matrix metalloproteinase-2 expression in rat aortic smooth muscle cells. Mol. Cell. Biochem..

[70] Lizarbe TR (2009). Nitric Oxide Induces the Progression of Abdominal Aortic Aneurysms through the Matrix Metalloproteinase Inducer EMMPRIN. Am. J. Pathol..

[71] Carrick D (2016). Myocardial hemorrhage after acute reperfused ST-segment-elevation myocardial infarction: Relation to microvascular obstruction and prognostic significance. Circ. Cardiovasc. Imaging.

[72] Adameova A (2017). Evidence of necroptosis in hearts subjected to various forms of ischemic insults. Can. J. Physiol. Pharmacol..

[73] Luedde M (2014). RIP3, a kinase promoting necroptotic cell death, mediates adverse remodelling aftermyocardial infarction. Cardiovasc. Res..

[74] Zhe-Wei S, Li-Sha G, Yue-Chun L (2018). The Role of Necroptosis in Cardiovascular Disease. Front. Pharmacol..

[75] Chen J (2019). The Neuroprotective Effects of Necrostatin-1 on Subarachnoid Hemorrhage in Rats Are Possibly Mediated by Preventing Blood–Brain Barrier Disruption and RIP3-Mediated Necroptosis. Cell Transplant..

[76] Moriwaki K, Chan FK-M (2013). RIP3: a molecular switch for necrosis and inflammation. Genes Dev..

[77] He BJ (2011). Oxidation of CaMKII determines the cardiotoxic effects of aldosterone. Nat. Med..

