# Supplementary material for: Bisphenol A induces coronary endothelial cell necroptosis by activating RIP3/CamKII dependent pathway
Source: Sci Rep. 2020 Mar 6;10:4190. doi: 10.1038/s41598-020-61014-1 (PMC7060177; doi:10.1038/s41598-020-61014-1)
Supplement: Supplementary file 1 — Supplementary information. [file 41598_2020_61014_MOESM1_ESM.pdf]

Supplementary Information

Bisphenol A induces coronary endothelial cell necroptosis by activating RIP3 /CaMKII dependent pathway.

Authors: Reventun P*, Sanchez-Esteban S*, Cook A*, Cuadrado I[+], Roza C*, Moreno-Gomez-Toledano R*, Muñoz C*, Zaragoza C[$], Bosch RJ*, Saura M*

Contents:

• Material and methods page 2
• Table 1 page 3
• Suplemental figure S1   page 4
• Suplemental figure S2   page 5
• Suplemental figure S3 page 6
• Suplemental figure S4 page 7
• Suplemental figure S5 page 8

Reagents and antibodies

General cell culture supplies were purchased from Lonza (Basel, Switzerland); calf serum was from Gibco(Waltham, Ma, USA). Matrigel, Cell recovery solution from BD Biosciences (San José, Ca, USA). Masson's trichrome staining kit EMD Millipore Corporation (Burlintong, Ma, USA), Master polymer plus detection system (peroxidase and DAB) were from Master diagnostic (Santander, Spain). DeadEnd™ Fluorometric TUNEL System was from Promega (Madison, WI,USA). Cell culture-grade gelatin, hematoxylin-eosin, Direct Red 80, Picric acid Saturated aqueous solution (1.3% in water) were from Sigma Aldrich (San Luis, MO, USA). Amersham ECL detection kit was from GE Healthcare Life Sciences (Barcelona, Spain). RIP3 siRNA and Control siRNA-A were from Santa Cruz Biotechnology (Santa Cruz, CA, USA). TRIzol reagent and Lipofectamine 2000 from Invitrogen Corporation (Carlsbad,CA, USA). RT-PCR kit, Complet mini and phosfo-stop were from Roche (Swtzerland).

A detail listing of the antibodies used through the study is provided bellow.

Antibody	Supplier	Reference	Dilution
**Alexa Fluor 488–conjugated wheat germ agglutinin**	Life Technologies	W11261	IF: 1-100
**Anti-Collagen I**	Abcam	ab34710	WB: 1-500
**Anti-TGFβ**	Santa Cruz	sc-130348	WB:1-500
**HRP-conjugated anti-Mouse secondary antibody**	Invitrogen	A16072	WB: 1-2000
**HRP-conjugated anti-Rabbit secondary antibody**	Invitrogen	31466	WB: 1-5000
**Anti-GAPDH**	Sigma	G8796	WB:1-1000
**Anti-Actin**	Sigma	A2066	WB:1-1000
**Anti-Fibrinogen**	Abcam	ab34269	IHQ: 1-50
**Anti-CD31**	Abcam	ab28364	IF:1-50
**Anti-MAC-2**	Santa Cruz	sc-18822	IHQ: 1-50
**Anti-Caveolin-3**	Abcam	ab30750	IF:1-50
**Rb pAb to 4 Hidroxinonenal**	Abcam	ab46545	IHQ: 1-50
**Anti-cd68**	Invitrogen	ma5-13324	IF: 1-50
**Anti-CCR2**	Thermo scientific	PA5-23037	IF:1-50
**Alexa fluor 647 conjugated Goat anti-Rabbit secondary antibody**	Abcam	ab150083	IF:1-500
**Alexa fluor 488 conjugated Goat anti-Rabbit secondary antibody**	Abcam	ab150081	IF:1-500
**Alexa fluor 647 conjugated Goat anti-mouse secondary antibody**	Abcam	ab150115	IF:1-500
**Anti-a-smooth Muscle actin**	Cell signaling	19245T	IF:1-200
**Anti- RIP3**	Santa Cruz	sc-374639	WB: 1-500<br>FC: 1-50
**Anti-IL10**	Abcam		IHQ:1-50
**Anti-CAMKII**	Santa Cruz	sc-9035	WB: 1-500
**Anti- pCAMKII**	Santa Cruz	sc-12886	WB: 1-500
**Anti-Caspase-3 p11 (C-6)**	Santa Cruz	sc-271759	IF:1-50
**Anti-Caspase 8**	Cell signaling	9746	WB: 1-500
**Anti PARP-1**	Santa Cruz	sc-7150	WB: 1-500

Table 1

qPCR

The qPCR conditions were Standard Cycling Mode (Primer Tm ≥60°C) first UDG Activation 50°C 2 min, then AmpliTaq® DNA Polymerase, UP Activation 95°C2 min, denature 95°C 15 sec and anneal/Extend 60°C 1 min.
The following primers were used:

TNF-a forward	5’ CAG CCG ATG GGT TGT ACC TT 3’
**TNF-a reverse**	5’ GGC AGC CTT GTC CCT TGA 3’
**CCL2 forward**	5’GTC TGT GCT GAC CCC AAG AAG3’
**CCL2 reverse**	5’TGG TTC CGA TCC AGG TTT TTA3’
**CCL7 forward**	5’CCA CAT GCT GCT ATG TCA AGA3’
**CCL7 reverse**	5’ACA CCG ACT ACT GGT GAT CCT3’
**CCL12 forward**	5’ATT TCC ACA CTT CTA TGC CTC CT3’
**CCL12 reverse**	5’ATC CAG TAT GGT CCT GAA GAT CA3’
**CCR2 forward**	5’TTA CAC CTG TGG CCC TTA TTT3’
**CCR2 reverse**	5’CTG AGT AGC AGA TGA CCA TGA C3’

Figure S1

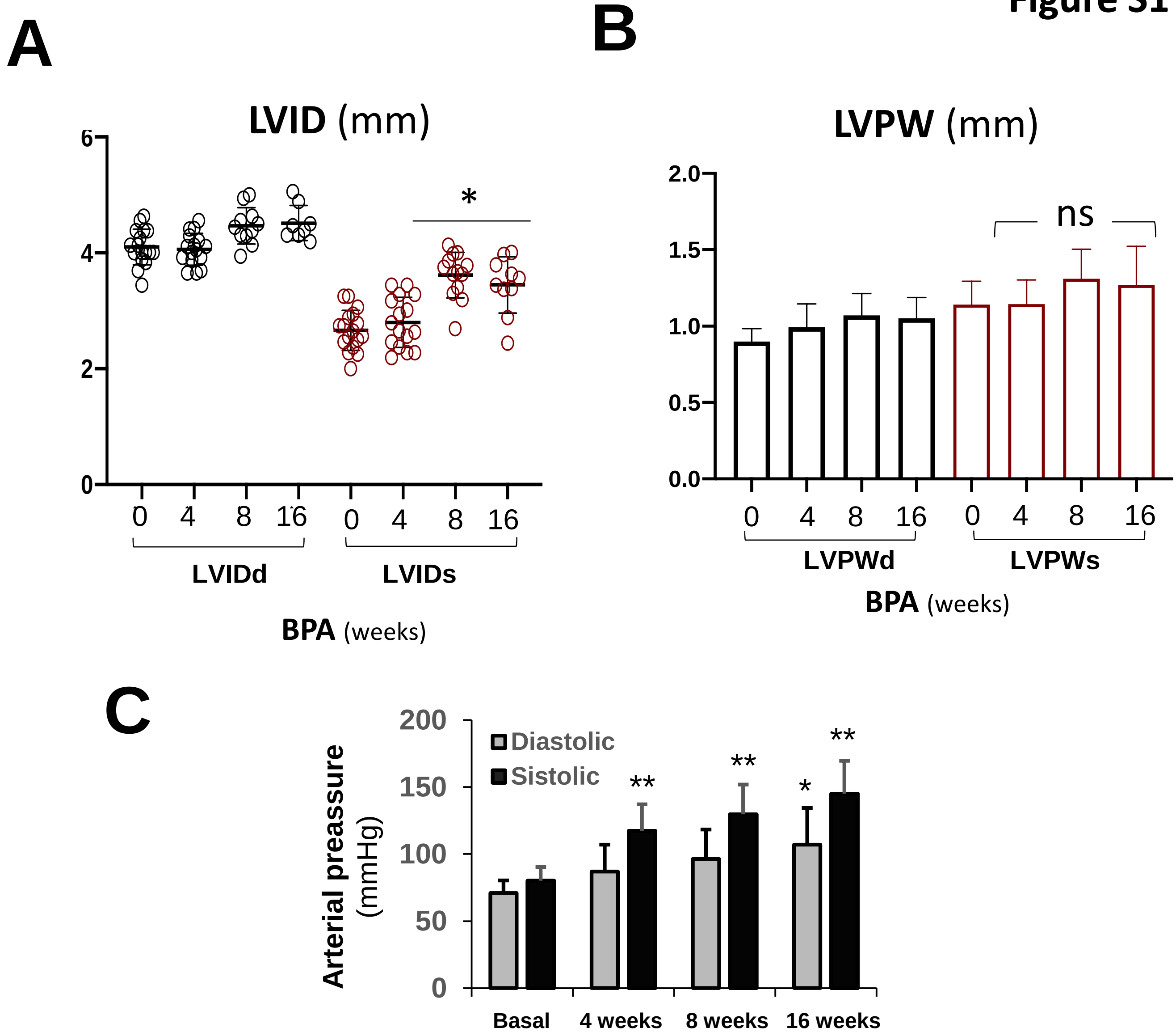

Figure S1 : BPA induces cardiac dysfunction and hypertension in mice. Mice were treated orally with BPA for 4, 8 and 16 weeks. Echocardiography was performed at basal conditions (time =0 weeks) and the indicated time points. **(A)** Left ventricular internal diameter (LVID) in diastole (LVIDd) and Systole (LVIDs).(n=8-10 mice per condition, *p< 0.05 vs LVIDs 0 weeks). **(B)** LV Posterior Wall thickness in diastole (LVPWd) and Systole (LVPWs). Bars represent mean ± SD. n=12 ;n.s : non significant **(C)** **A**rterial blood pressure measurement in mice either basal conditions (0 weeks) and after 4, 8 and 16 weeks of BPA. *p< 0.05 and ** p< 0.01 n=20 per condition**.**

Figure S2

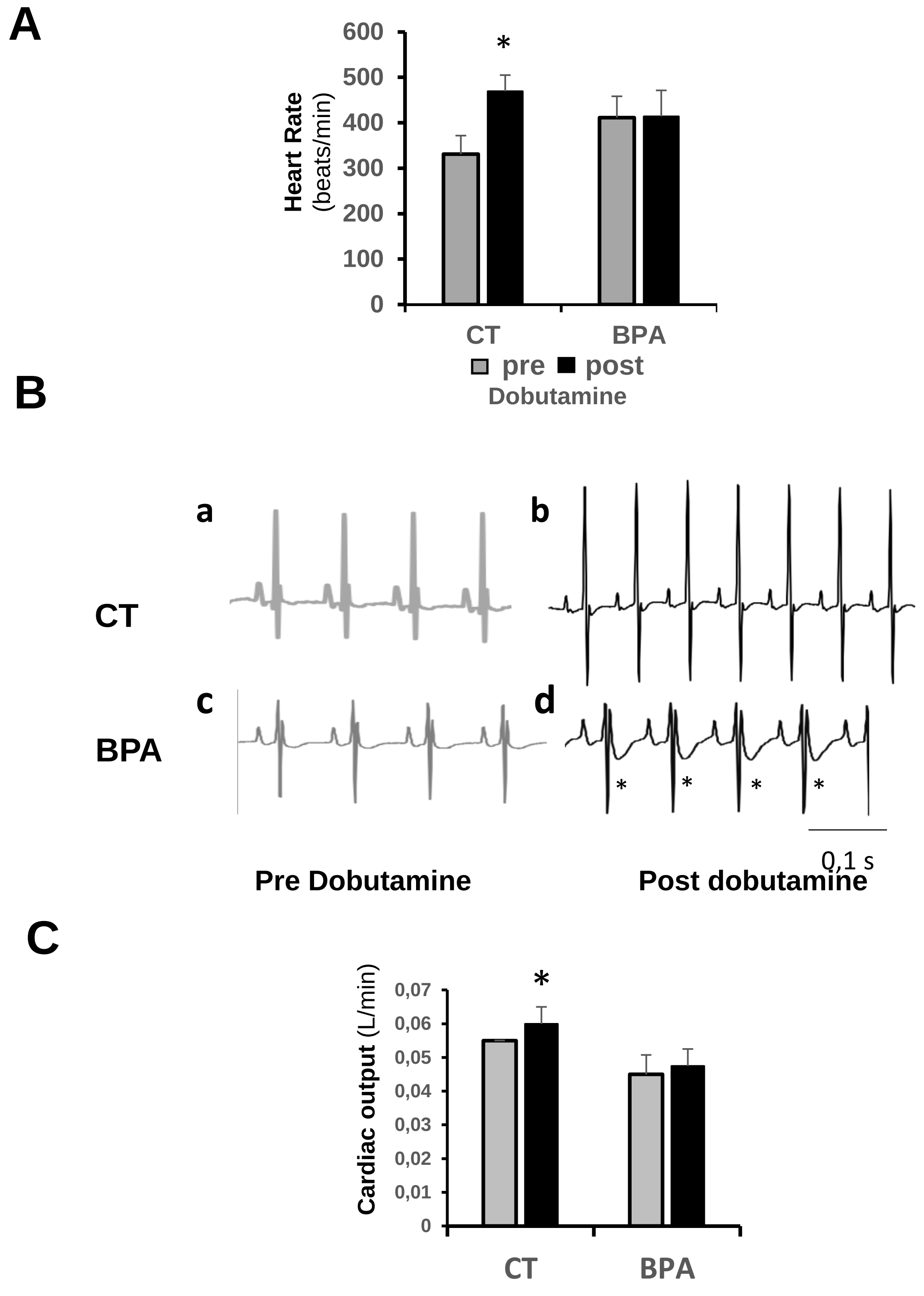

Figure S2 : BPA produces abnormal dobutamine stress test results. CT and 16 weeks BPA treated were challenged with i.p. injection of 3mg/k of dobutamine while recording cardiac activity by EGC or echocardiogram for 15 min. (**A)** Heart rate as detected by echocardiography showing the lack of positive chronotropic response to Dobu in BPA mice. (n =4 per group, * $p < 0.05$). **(B)** Typical ECG records from CT **a** and **b** and BPA 16 weeks **c** and **d** ilustrating **a**) normal synus rihythm **b)** increased heart rate after five min dobutamine stimulation, **c)** increased PR duration pre dobutamine **d)** ST depression following dobutamine challenge (marked with an asterisk) **(C)** Cardiac output in CT and BPA treated animal before grey bars and after dobutamine injection black bars n=6 animals per condition. *p< 0.05 vs CT pre dobutamine

Figure S3

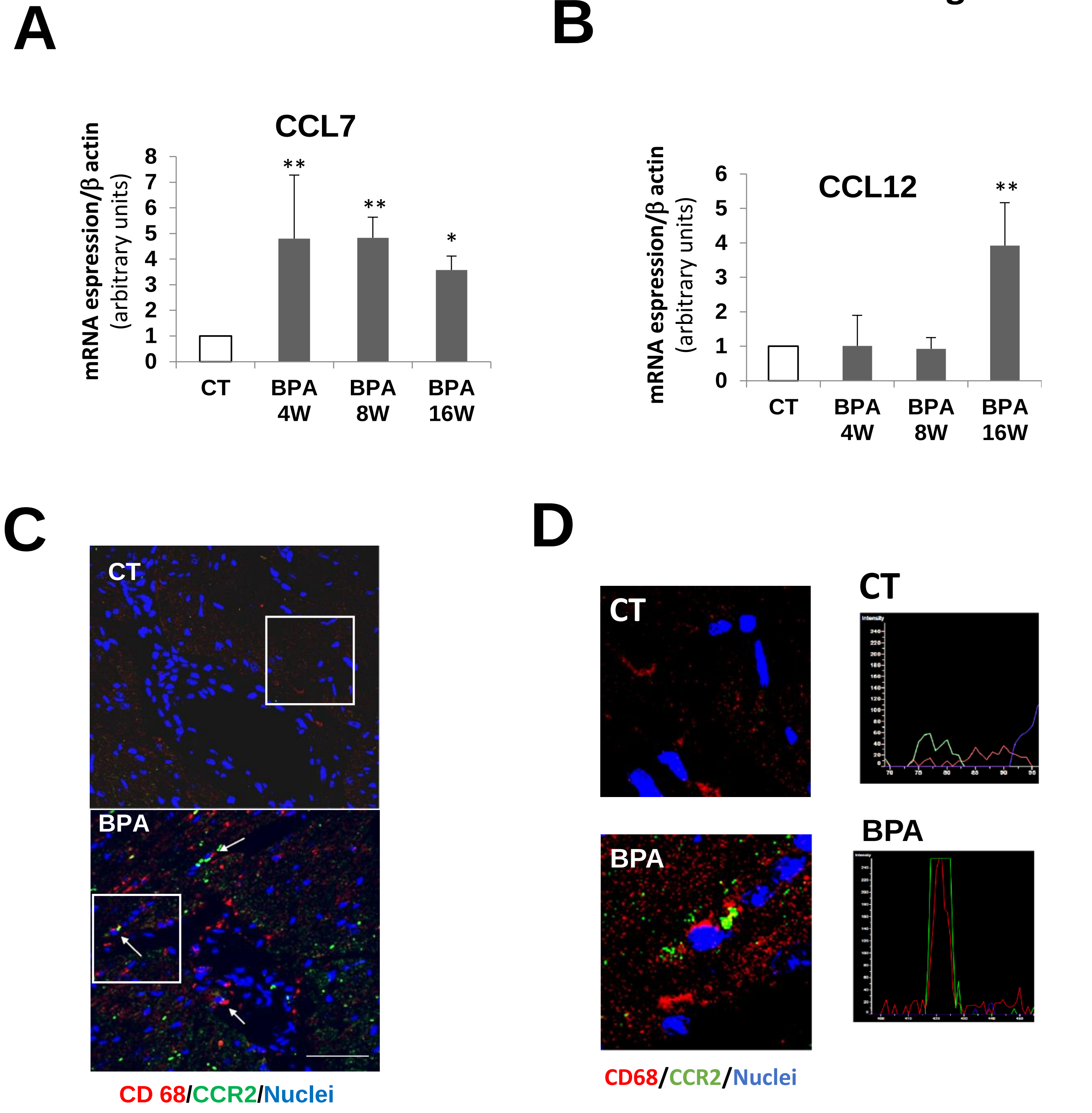

Figure S3 :BPA induces a chronic inflammatory response. RT- qPCR of CT and 4, 8 and 16 weeks BPA treated mice showing cardiac mRNA expression of **(A)** CCL7 and **(B)** CCL12 (n=8 per condition with triplicates in each determination, $^*p<0.05$; $^{**}p< 0.01$ vs CT). **(C)** Representative confocal images of heart sections of CT and 8 weeks BPA treated mice (n = 5-8 per group) immunostained for CD68 (red) and CCR2 (green). Nuclei were labeled with Hoechst (blue). Scale bar = 25μm. Arrows point to cells CD68+/CCR2+. ROIs marked in white squares indicate the regions of colocalization which are shown enlarged in **D)** 4x magnification. Representative histograms of CD68/CCR2 colocalization are shown on the right panel. The images presented here are representative of at least five different hearts per condition.

Figure S4

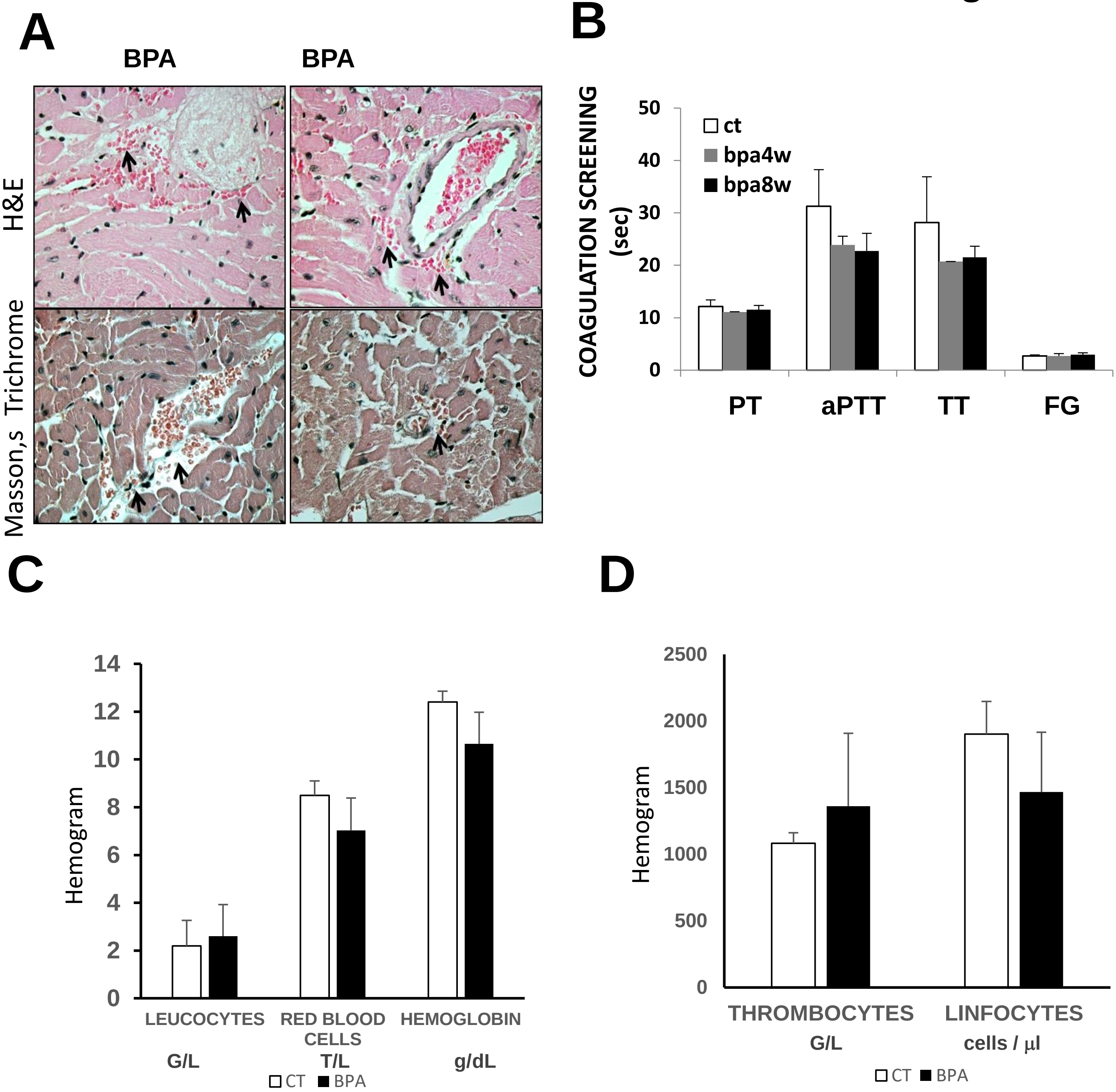

Figure S4. BPA does not induce a coagulation defect. A) Cardiac hemorrhagic lesions as detected by H&E and Masson's trichrome staining of BPA treated with 0,04 and 0,4 µM for 8 weeks. Blood parameters in CT and BPA treated mice. **B)** Coagulation screening CT n= 12; BPA 4 weeks n=10, 8 weeks n=8. PT (Prothrombin time); aPTT (Activated partial thromboplastin time) ; TT (Thromboplastin time) ; FG (Fibrinogen)(g/L) **C)** and **D)** Hemogram performed in CT and BPA treated mice 16 weeks.

Figure S5

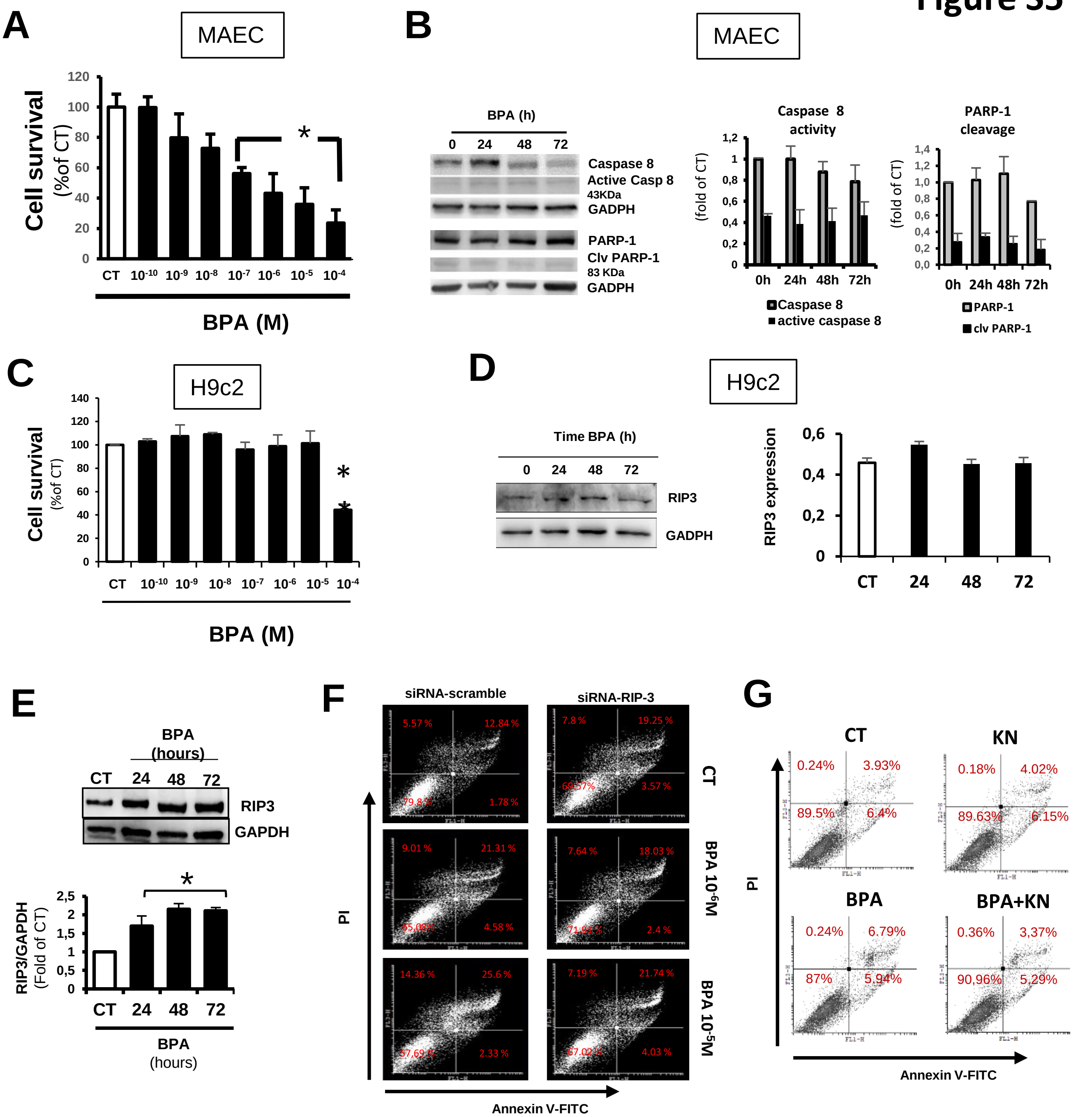

Figure S5: BPA enhances endothelial cell death. (A) Mouse aortic endothelial cells **(**MAEC) were treated for 24h with vehicle (CT) or BPA at different concentrations (10^{-10} to 10^{-4} M) 24h, and MTT assays were performed. Data represent cell survival expressed as a percentage of CT. (n=3 with triplicates per condition *p<0.05 vs. CT;) **(B)** MAEC were treated with 10^{-6}M at different time points and caspase 8 activity and PARP-1 cleavage, as a surrogate marker of caspase 3 activation, were measured by western blot. GADPH was used as loading control. Data represent media ± SD of at least 4 independent experiments; **(C)** Rat neonatal cardiomyocyte (H9c2) were treated for 24h with vehicle (CT) or BPA at different concentrations (10^{-10} to 10^{-4} M) 24h, and MTT assays were performed as in A). Data represent cell survival expressed as a percentage of CT. n=3 *p<0.05 vs. CT **(D)** H9c2 cells were treated with BPA 10^{-6}M for 24, 48 and 72h and RIP 3 expression levels studied by western blot. A representative immunoblot is shown. Data shown as mean ± SD (n=3); **E)** Immunoblot analysis of RIP3 expression in MAEC treated with 10^{-6} M BPA for 24, 48, and 72 h. GAPDH was used as a loading control. A representative immunoblot is shown. Data are shown as mean ± SD (n=4 with duplicates in each condition), *p<0.05 vs. CT; **F)** Dot plot representative of flow cytometry analysis of MAEC transfected with RIP3-specific siRNA (Si RIP3) or non-silencing siRNA scramble (si Sc) and treated with and without BPA at 10^{-6}M and 10^{-5}M for 24h. Cells were stained with annexin V-FITC and propidium iodide. **G)** Dot plot representative of flow cytometry analysis of MAEC treated with Kn-93 (an inhibitor of Ca-CamKII activity) at 10^{-6}M and treated with and without BPA at 10^{-6}M for 24h. Cells were stained with annexin V-FITC and propidium iodide.
